# Discovery of enhanced lattice dynamics in a single-layered hybrid perovskite

**DOI:** 10.1126/sciadv.adg4417

**Published:** 2023-08-16

**Authors:** Zhuquan Zhang, Jiahao Zhang, Zi-Jie Liu, Nabeel S. Dahod, Watcharaphol Paritmongkol, Niamh Brown, Alexia Stollmann, Woo Seok Lee, Yu-Che Chien, Zhenbang Dai, Keith A. Nelson, William A. Tisdale, Andrew M. Rappe, Edoardo Baldini

**Affiliations:** ^1^ Department of Chemistry, Massachusetts Institute of Technology, Cambridge, MA 02139, USA.; ^2^ Department of Chemistry, University of Pennsylvania, Philadelphia, PA 19104, USA.; ^3^ Department of Chemical Engineering, Massachusetts Institute of Technology, Cambridge, MA 02139, USA.; ^4^ Department of Materials Science and Engineering, Massachusetts Institute of Technology, Cambridge, MA 02139, USA.; ^5^ Department of Physics, The University of Texas at Austin, Austin, TX 78712, USA.

## Abstract

Layered hybrid perovskites exhibit emergent physical properties and exceptional functional performances, but the coexistence of lattice order and structural disorder severely hinders our understanding of these materials. One unsolved problem regards how the lattice dynamics are affected by the dimensional engineering of the inorganic frameworks and their interaction with the molecular moieties. Here, we address this question by using a combination of spontaneous Raman scattering, terahertz spectroscopy, and molecular dynamics simulations. This approach reveals the structural dynamics in and out of equilibrium and provides unexpected observables that differentiate single- and double-layered perovskites. While no distinct vibrational coherence is observed in double-layered perovskites, an off-resonant terahertz pulse can drive a long-lived coherent phonon mode in the single-layered system. This difference highlights the dramatic change in the lattice environment as the dimension is reduced, and the findings pave the way for ultrafast structural engineering and high-speed optical modulators based on layered perovskites.

## Teaser

A comparison of lattice dynamics in single- and double-layered hybrid perovskites is presented by using advanced spectroscopies.

## INTRODUCTION

Over the past decade, two-dimensional hybrid perovskites (2DHPs) have emerged as natural quantum-well-like semiconductors with marked light absorption, large luminescence quantum yield (*1*, *2*), and strong exciton binding energy (*3*, *4*). Unlike their 3D counterparts, 2DHPs also show wider chemical variability and structural diversity, as their composition can be tuned by altering organic spacer cations, inorganic networks, and the number of octahedral layers (*5*–*8*). This richness may also lead to a plethora of emergent properties including ferroelectricity (*9*, *10*), spin selectivity (*11*), and multifunctionality (*12*). Although substantial efforts have been made to exploit this versatility by dimensional tailoring, it is an ongoing task to establish the structure-function relationships in these materials. Key to this goal is an understanding of the interplay between the inorganic lattice framework and the organic cations (*13*, *14*). Previous mechanistic studies have suggested that the hybrid lattice features notable anharmonicity and polarizability, in conjunction with structural disorder (*15*–*17*). However, it remains an open question whether these properties persist to the single-layer limit when the octahedral framework does not fully resemble the lead-halide backbone of the bulk perovskite structure.

Here, we present a joint experimental-theoretical study aimed at uncovering the origin of the dynamic structural complexity in 2DHPs. By means of steady-state and ultrafast spectroscopy experiments, we identify unique fingerprints that distinguish the structural dynamics of the hybrid lattices in the crossover between quasi-2D and 2D. We observe that the collective motion of the octahedral cages is substantially enhanced as the number of layers per repeating unit is altered from two to one. Our results are rationalized via molecular dynamics (MD) calculations, which provide an atomic-level understanding of the hybrid lattice in and out of equilibrium.

## RESULTS

**Fig. 1.**
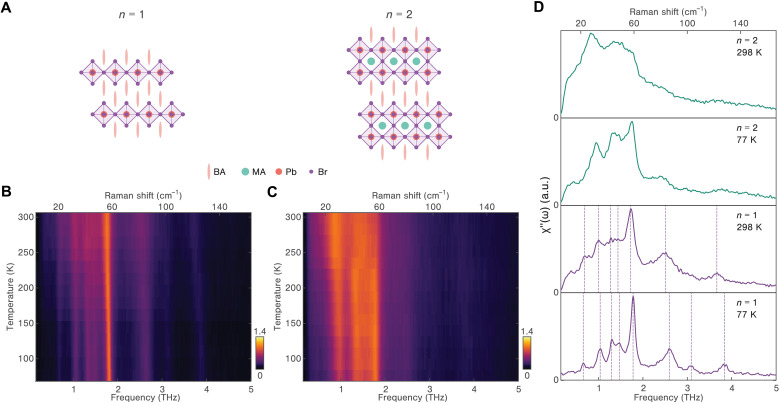
Crystal structure and spontaneous Raman responses of 2DHPs. (**A**) Schematic illustration of crystal structure for both single-layer (*n* = 1) and double-layer (*n* = 2) bromide perovskites. BA, butylammonium; MA, methylammonium; Pb, lead; Br, bromide. (**B** and **C**) Temperature-dependent Raman spectra of *n* = 1 and *n* = 2 2DHPs from 77 to 298 K. (**D**) Selected Raman spectra of *n* = 1 (bottom) and *n* = 2 (top) 2DHPs at 77 K (bright purple and green) and 298 K (light purple and green). All the phonon modes are indicated by dashed lines. a.u., arbitrary units.

We focus on two prototypical 2DHPs which differ in the number of corner-sharing octahedral layers *n*: (BA)_2_PbBr_4_ (single-layer, *n* = 1) and (BA)_2_MAPb_2_Br_7_ (double-layer, *n* = 2). These 2DHPs, with Ruddlesden-Popper structures, crystallize in *Pbca* and *Cmc2_1_* space groups, respectively. As shown in Fig. 1A, *n* = 1 2DHP only contains organic spacer ligands (BA, butylammonium) that separate the octahedral layers, while the double-layered 2DHP consists of additional A-site cations (MA, methylammonium) that occupy cuboctahedral pockets formed by eight octahedra.

As a first step in the study of the lattice dynamics in thermal equilibrium, we monitor low-energy collective responses and structural disorder via high-resolution spontaneous Raman scattering (*18*). We map the Raman spectra of both 2DHPs across a wide range of temperatures as shown in Fig. 1B (*n* = 1) and Fig. 1C (*n* = 2). For *n* = 1 2DHP, the Raman spectra are characterized by eight well-defined peaks that include a dominant mode at 1.8 THz (*19*). In contrast, the Raman data of *n* = 2 2DHP exhibit broad features at all temperatures. In Fig. 1D, we provide a detailed comparison by selecting the Raman spectra of both materials at room temperature and at 77 K. The room temperature spectra for both *n* = 1 and *n* = 2 crystals show a prominent spectral continuum below 5 THz, which is reminiscent of quasi-elastic peaks due to disorder (*20*–*22*); however, the 1.8 THz peak in the *n* = 1 compound stands out from the underlying continuum. When the temperature is decreased to 77 K, the continuum background contribution to the *n* = 1 Raman response is markedly reduced, and several additional modes become distinct. In contrast, the low-temperature spectrum of *n* = 2 2DHP exhibits persistent broad features, suggesting the coexistence of phonon peaks and structural disorder. From the steady-state Raman data, we establish a thermal equilibrium view of the structural complexity in 2DHPs and conclude that the structural disorder is considerably reduced in the *n* = 1 compound with single octahedral planes.

On the basis of these Raman data, we also find that the behavior of bromide 2DHPs stands in stark contrast to that of iodide 2DHPs. While the latter has been reported to undergo various low-temperature phase transitions by previous works (*22*–*25*), no structural phase transition is observed in the *n* = 1 and *n* = 2 bromide 2DHPs investigated in this study. This finding is further supported by temperature-dependent powder x-ray diffraction data included in the Supplementary Materials, establishing a benchmark for comparing octahedral dynamics in the transition from double-layer to single-layer structure.

**Fig. 2.**
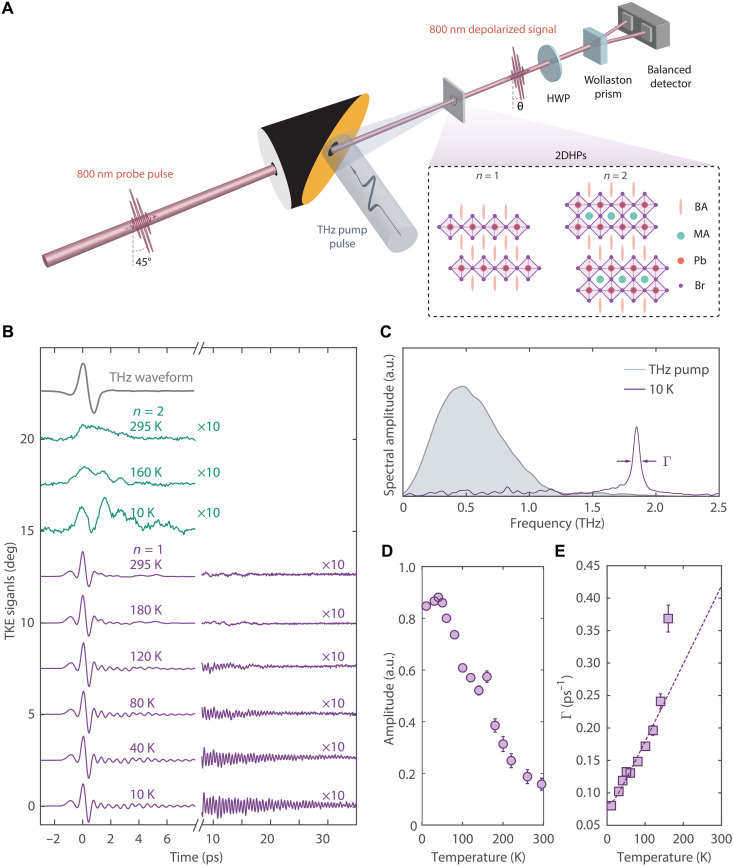
THz Kerr effect spectroscopy measurements. (**A**) The single-cycle THz pump is focused on 2DHP single crystal to induce a nonlinear response. The time-delayed 800 nm probe pulse is polarized at 45° relative to the vertical THz polarization and the transiently depolarized signal is measured by a balanced detection scheme. HWP, half-wave plate. (**B**) Time-resolved THz-Raman signals for both *n* = 1 (purple) and *n* = 2 (green) 2DHPs at various temperatures. The TKE signals for *n* = 2 2DHP and the long-lived oscillations in the *n* = 1 sample are magnified by 10. Data are vertically shifted for clarity. The THz pump waveform (gray) is also shown at the top. (**C**) Fourier transform analysis of the oscillatory signal in *n* = 1 sample at 10 K in (A) reveals a single peak at 1.8 THz, which is above the spectral content of the incident THz pulse (gray area). (**D**) Temperature dependence of the *n* = 1 1.8 THz mode amplitude. The amplitude becomes nonzero below 200 K and increases monotonically as the temperature is decreased. (**E**) The 1.8 THz mode decay rate as a function of temperature below 200 K. The dashed blue curve is a fit to an anharmonic decay model (see note S4). The error bars in (D) and (E) represent the 95% confidence interval.

Next, we track the time evolution of the lattice response at ultrafast timescales to attain more insights into the structural dynamics and separate the collective modes from the dynamic disorder (*26*, *27*). Optical Kerr effect spectroscopy (*28*) was used to investigate lattice (*29*) and molecular reorientation dynamics (*30*) in hybrid perovskites, but the signals were substantially affected by the nonlinear light propagation effect (*31*). While transient absorption spectroscopy has previously revealed coherent lattice dynamics in both 3D and 2D perovskites (*32*–*35*), these studies relied on the fact that the phonon modes couple to the excited electronic states, which added another layer of complexity and led to large detection background. In our study, we use THz field-induced Kerr effect (TKE) spectroscopy (*36*) to monitor the lattice behavior in real time by resolving the nonlinear polarizability dynamics (see Fig. 2A). This technique provides several advantages. First, the THz frequency photons match the natural energy scale of low-energy vibrations of the inorganic frameworks and therefore only perturb the lattice degrees of freedom. Second, by using the slowly varying electric field of THz pulses, we can also strongly displace the cloud of electrons bound to the nucleus and induce giant polarizability responses (*37*) that may not be accessible with pump pulses in the optical frequency range (see note S3).

Figure 2B shows the TKE data recorded at several temperatures for both 2DHPs. To minimize the potential misinterpretation of Kerr effect signals due to the temporal walk-off between the pump and probe pulses within the anisotropic media (*38*, *39*), we selected samples with the same thickness (i.e., ~100 μm). For the *n* = 2 sample at room temperature (green curve), only nonoscillatory signals are observed after the initial electronic response induced by the THz pulse. When the temperature is lowered below 160 K, oscillations emerge but only last for a few cycles, less than 5 ps. These observations indicate that in *n* = 2 2DHP, any excited phonon mode loses its coherence quickly, consistent with the observation of broad features in the steady-state Raman spectra. In contrast, TKE signals from the *n* = 1 2DHP exhibit notably different behavior. First, the response is 10 times larger than its *n* = 2 counterpart. This distinction indicates the presence of enhanced polarizability at THz frequencies, as the two materials have similar dielectric properties at the probe photon energy (see note S2). Second, the initial response shows a bipolar character (*40*, *41*), signaled by the two lobes with opposite signs that do not follow the incident THz waveform. Although differences in the lineshape of the TKE signals may be explained by the nonlinear dispersion effects (*31*, *38*, *39*), a notable difference in the *n* = 1 system lies in the immediate appearance of a long-lived sinusoidal modulation following the initial electronic response in the *n* = 1 system. The oscillation frequency of 1.8 THz (see Fig. 2C) corresponds to the most prominent phonon mode in the equilibrium Raman spectra. Even at room temperature, this coherent phonon response remains detectable for the first few picoseconds. Decreasing the temperature results in a simultaneous increase in the oscillation amplitude and decay time (see Fig. 2, D and E), which is a manifestation of the suppressed structural disorder and reduced anharmonic decay of the excited mode (see note S4). Notably, at 10 K, this Raman coherence can persist up to 30 ps without entirely damping to zero.

**Fig. 3.**
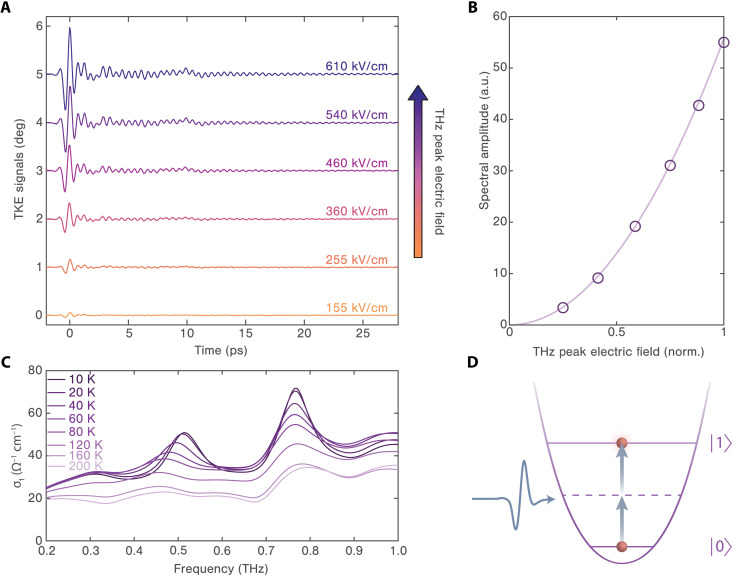
Generation mechanism of the long-lived phonon oscillation in the *n* = 1 2DHP. (**A**) TKE signals at 10 K are shown as a function of THz pump electric field strength. Data are vertically shifted for clarity. (**B**) Fourier transform analysis of signals in (A) shows the spectral amplitudes of the mode as a function of THz pump electric field strength. The light purple line represents a quadratic fit. (**C**) The real part of THz conductivity in the *n* = 1 sample as a function of temperature is measured by time-domain THz spectroscopy. The low-temperature curves show two resonance peaks corresponding to two infrared-active phonon modes. (**D**) The sum frequency of two incident THz electric-field components is resonant with the transition between the ground and the first excited states to drive the Raman-active mode.

To identify the underlying mechanism that drives this long-lived coherent phonon response, we obtain the THz field-dependent TKE signals for *n* = 1 2DHP at 10 K. As shown in Fig. 3A, the time-domain oscillatory responses increase monotonically with the THz field strengths. Fourier transformation of the oscillatory parts of the TKE signals shows that the spectral amplitude scales as the square of the pump electric field (see Fig. 3B). This observation indicates that the Raman mode at 1.8 THz is driven through a second-order interaction with the THz electric pump field. Such a nonlinear excitation process can proceed through two distinct pathways, which are ionic or photonic in nature (see note S3). (*42*) In the ionic scenario, the THz electric field drives a dipole-active mode and the nonlinear excitation of the Raman mode is mediated by anharmonic phonon-phonon coupling (*43*, *44*), whereas in the photonic mechanism, the THz electric field drives the Raman mode directly through the nonlinear polarizability (*45*–*47*). Since the frequency of the Raman mode is well above the bandwidth of the incident THz pulse (see Fig. 2C), we can also exclude the scenario of impulsive stimulated Raman scattering (*45*, *48*) or impulsive ionic Raman scattering (*49*–*51*) in which difference-frequency components of the photon or phonon field drives the Raman mode. Rather, the sum-frequency excitation pathway should be responsible for the observed Raman excitation. For such a process to be ionic, there must be an infrared-active phonon mode directly driven by the THz pump, whose phonon frequency is ideally at half of the Raman mode frequency (i.e., Ω_IR_ = Ω_R_/2 ~ 0.9 THz). Since the crystal is centrosymmetric, only Raman-active modes produce transient birefringent signals, and therefore, oscillations in the TKE responses do not reflect coherent excitation of infrared modes directly driven by the THz field (*52*, *53*). To provide additional evidence, we apply time-domain THz spectroscopy, which directly measures dipole-allowed transitions in the THz frequency range. Figure 3C shows the real part of THz conductivity at various temperatures below 200 K, which reveals two infrared-active modes (i.e., at 0.5 and 0.75 THz) emerging at low temperatures. Yet, there is no infrared-active phonon mode at 0.9 THz that fulfills the resonance condition of sum-frequency ionic Raman excitation, and the Raman coherence is also observed at elevated temperatures where the infrared phonons are substantially damped. Furthermore, it was previously established that the ionic excitation mechanism would lead to beat signals in the time domain, resulting from the mutual exchange of energy between the driven infrared-active and Raman-active phonon modes (*44*). In our data, we only observe an exponential decay of the Raman coherence (see note S4 and fig. S7). This rules out any possibility that the 1.8 THz Raman-active mode is driven by anharmonic coupling to an excited infrared-active phonon mode. Therefore, we can conclude that the observed coherent collective response is generated by sum-frequency photonic excitation (see Fig. 3D) (*47*, *54*).

**Fig. 4.**
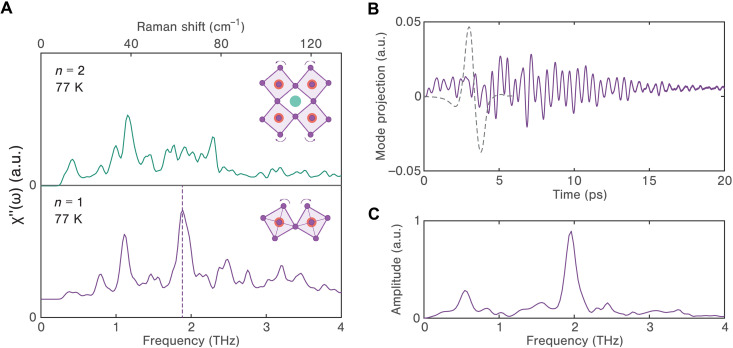
MD simulation results. (**A**) Simulated Raman spectra of both *n* = 1 (bottom) and *n* = 2 (top) 2DHPs at finite temperature (77 K). The insets depict the corresponding octahedral tilting and rotation motion for *n* = 1 and *n* = 2 2DHPs. (**B**) MD simulation trajectory projection onto the 1.9 THz mode (solid purple) along with the input THz electric field waveform (dashed gray). (**C**) Fourier transform of the trajectory projection in (B).

To gain deeper insight into the nature of both thermal and coherent dynamics, we conduct ab initio MD simulations for a √ 2 × √ 2 × 2 cell. To simulate equilibrium states, we use the canonical (NVT) ensemble for calculating the spontaneous Raman responses. Figure 4A displays the simulated Raman spectra for both *n* = 1 and *n* = 2 2DHPs at 77 K, which agree well with the experimental data at the same temperature. The Raman spectrum of the *n* = 1 2DHP exhibits a distinctive peak at around 1.9 THz, which closely corresponds to the frequency of the most prominent phonon mode observed in the experimental data (1.8 THz). Through real-space analysis of the MD simulations (see note S5), we identify this mode as the bending and twisting of the octahedral cages in the single-layer inorganic framework. In contrast, the *n* = 2 2DHP exhibits a more disorder-dominated Raman response. The computed autocorrelation functions and the spectral decomposition of molecular reorientation dynamics underscore dynamic disorder introduced by additional MA organic cations and their consequential role in establishing more intricate chemical environments within the lattice (refer to figs. S10 and S11). MA molecules situated within the cages can dynamically deviate from the planar alignment, resulting in disparate responses between the top and bottom octahedra and introducing additional scattering paths for octahedral coherences. Moreover, since the two adjacent layers of lead bromide octahedra in the *n* = 2 system are bonded to each other, with octahedral distortion introduced by the MA cations, the lattice responses are substantially more anharmonic compared to the *n* = 1 system. This is consistent with previous findings showing that stiffness is reduced in the *n* = 2 2DHP (*55*). These multifaceted sources of disorder naturally explain why the Raman spectra display broader features for the *n* = 2 system. We also investigate the nonlinear THz light-matter interaction in the *n* = 1 2DHP. To capture coherent dynamics rather than the thermal fluctuation of the lattice response, we conduct MD simulations in the microcanonical ensemble (NVE). We apply the experimentally measured THz electric field waveform to our system, setting its polarization in the plane of the octahedral layers. We then project the MD trajectory into the eigenmode basis calculated from density functional perturbation theory (DFPT) (*56*–*58*). As shown in Fig. 4B, we find that the driving THz field generates a long-lived oscillatory response that does not decay to 0 up to 20 ps. The Fourier transform spectrum in Fig. 4C reveals a sharp peak centered at around 1.9 THz, in excellent agreement with our experimental data.

## DISCUSSION

Our spectroscopic measurements combined with the MD simulations establish that the single-layered hybrid perovskite features a giant polarizable lattice response that does not exist in the double-layered perovskite counterparts. This finding highlights the use of tailored THz light excitation to study hybrid lattices exhibiting a complex interplay of molecular and ionic dynamics. From the fundamental point of view, this approach can be applied to explore many other structurally complex materials, including artificially engineered heterostructures and moiré superlattices, and opens the door to desirably controlling their emergent properties and unique functionalities with light (*59*). Given that our sample thickness is ∼100 μm, the estimated modulation depth of the THz field-induced polarization rotation is ∼2 dB/mm at room temperature (see note S6). Although we only demonstrate the polarization modulation for light of a single wavelength (800 nm), we expect that similar results will hold for a broad range of wavelengths below the material’s bandgap (i.e., >400 nm). For these reasons, we believe that 2DHPs are promising candidates for achieving all-optical, broadband refractive modulators at high speeds with tailored THz stimuli (*60*), offering prospects for the development of advanced optical devices (*61*).

## MATERIALS AND METHODS

### Synthesis of lead-bromide 2DHPs

Crystals of lead-bromide 2DHPs were synthesized by slow-cooling crystallization (*62*) following a previously reported procedure (*8*). First, a solution of lead (II) bromide (PbBr_2_) was prepared by dissolving PbO [99.9 + %, (trace metal basis) <10 microns, powder; ACROS Organic] in an aqueous hydrogen bromide solution (HBr; ACS reagent, 48%; MilliporeSigma). Then, a small volume of butylamine was added to the PbBr_2_ solution to form a white precipitate of (BA)_2_PbBr_4_ (single-layer, *n* = 1). To prepare (BA)_2_MAPb_2_Br_7_ (double layer, *n* = 2), a solution of MA bromide (MABr) salt was prepared in a separate vial by dissolving the salt in an aqueous HBr solution. The MABr solution was subsequently added to the solution of (BA)_2_PbBr_4_ to form a (BA)_2_MAPb_2_Br_7_ solution. Next, the solutions of (BA)_2_PbBr_4_ and (BA)_2_MAPb_2_Br_7_ were further diluted by additional volumes of HBr before being heated to 130°C until they became clear. After that, they were allowed to cool slowly to room temperature inside a thermos filled with hot sand at 110°C to induce crystallization. Crystals were then collected by suction filtration and dried under reduced pressure for at least 12 hours. The quantities of reagents used can be found in Table S1. The crystals grown via our method contains single-crystalline domains much larger than the optical probe size, as confirmed by polarized light imaging. To demonstrate this, we have included representative polarized light micrographs in the Supplementary Materials, as shown in Fig. S4.

### High-resolution spontaneous Raman scattering

Steady-state Raman spectra were collected in a backscattered geometry using a home-built micro-Raman instrument. Samples were housed within an optical cryostat (Janis ST500, fused quartz window) mounted to an inverted Nikon microscope (60×, 0.6 numerical aperture objective), and kept under vacuum during all measurements. A 785 nm narrowband continuous wave excitation source was filtered from undesirable amplified spontaneous emission using a series of cleanup filters (laser and filters from Ondax). The Rayleigh line was minimized by passing the collected signal through a set of volume holographic grating notch filters (from Ondax) before being dispersed in a 0.5 m focal length spectrograph (SP-2500, Princeton Instruments) using a 1200 g/mm and 750 nm blazed grating. The resulting spectrum was imaged with a cooled charge-coupled device camera (Princeton Instruments Pixis) with a typical signal integration time of 15 to 30 s. The Rayleigh notch filters, centered at 0 cm^−1^, have a full attenuation bandwidth of ±10 cm^−1^. The overall spectral resolution of the instrument is 0.9 cm^−1^. The spectra were calibrated by comparison to the longitudinal optical phonon position of a CdSe standard.

### TKE spectroscopy

The majority of the output of a 1 kHz Ti:Sapphire laser amplifier (800 nm, 12 mJ, 35 fs; Coherent Legend Elite Duo) was chopped at 500 Hz and used to generate single-cycle THz pulses via an optical rectification process with a tilted pulse front (*63*). The THz pulse was collected and focused by a pair of 90° off-axis parabolic mirrors. The remainder of the laser output was attenuated and used as a probe pulse that was focused along with the THz pulse onto the sample inside the cryostat. In the TKE experiment, the 800 nm probe pulse polarized at 45° relative to the polarization of the THz pulse was transmitted through the sample. The transmitted probe pulse was depolarized by the THz field-induced anisotropic responses, resulting in transient birefringence. The signal was measured by a pair of balanced photodiodes after a half-wave plate and a Wollaston prism.

### Time-domain THz spectroscopy

In time-domain THz spectroscopy experiments, the THz field was attenuated by a pair of wire-grid polarizers so that the measured signals were in the linear response regime. The transmitted THz waveform was focused into a ZnTe crystal and was overlapped with a gate pulse at 800 nm for the electro-optic sampling with a single-shot detection method (*64*). We determined the frequency-dependent complex transmission coefficient by comparing the THz electric field through the sample to that through a reference aperture of the same size. From the measured complex transmission coefficient, we numerically extracted the real and imaginary parts of the dielectric permittivity as a function of frequency.

### MD simulation

The density functional theory calculations were performed with Perdew-Burke-Ernzerhof exchange functional (*65*) using a plane-wave basis set with energy cutoff set at 50 rydbergs. We used the Opium software package (*66*) to generate norm-conserving pseudopotentials (*67*). The Raman spectrum was calculated via an MD approach as described before (*68*–*70*)

$$I_{ij}(\omega )=\frac{\omega }{1-\exp\left(-\frac{h\omega }{K_{B}T}\right)}\int {\left\langle \alpha_{ij}(\tau )\alpha_{ij}(t+\tau )\right\rangle }_{\tau }e^{-i\omega t}dt$$ (1)

where *I_ij_* (ω) is the Raman scattering intensity at frequency ω, and α*_ij_* is the electronic polarizability tensor obtained from DFPT (*56*–*58*). The polarizability α*_ij_*(*t*) was calculated on the basis of snapshots of MD trajectories. We used the Wiener-Khinchin theorem to calculate the autocorrelation function of the Raman susceptibility (*71*, *72*). The MD simulation is first equilibrated for 10 ps, and then sampled every 100 fs with a total 40 ps time window. The MD and Raman simulation was conducted using Quantum-ESPRESSO (*73*, *74*) and the temperature was controlled with the Nosé-Hoover thermostat (see note S3) (*75*, *76*).

## Supplementary Materials

### This PDF file includes:

Figs. S1 to S11 Table S1 Legends for movies S1 to S5 References

## References

[1] X. Gong, O. Voznyy, A. Jain, W. Liu, R. Sabatini, Z. Piontkowski, G. Walters, G. Bappi, S. Nokhrin, O. Bushuyev, M. Yuan, R. Comin, D. M. Camant, S. O. Kelley, E. H. Sargent, Electron–phonon interaction in efficient perovskite blue emitters. Nat. Mater. 17, 550–556 (2018). 29760510 10.1038/s41563-018-0081-x

[2] G. Grancini, M. K. Nazeeruddin, Dimensional tailoring of hybrid perovskites for photovoltaics. Nat Rev Mater 4, 4–22 (2019).

[3] M. D. Smith, B. A. Connor, H. I. Karunadasa, Tuning the luminescence of layered halide perovskites. Chem. Rev. 119, 3104–3139 (2019). 30689364 10.1021/acs.chemrev.8b00477

[4] C. M. Mauck, W. A. Tisdale, Excitons in 2D organic–inorganic halide perovskites. Trends in Chem. 1, 380–393 (2019).

[5] X. Li, J. M. Hoffman, M. G. Kanatzidis, The 2D halide perovskite rulebook: How the spacer influences everything from the structure to optoelectronic device efficiency. Chem. Rev. 121, 2230–2291 (2021). 33476131 10.1021/acs.chemrev.0c01006

[6] E. Shi, Y. Gao, B. P. Finkenauer, A. H. Coffey, L. Dou, Two-dimensional halide perovskite nanomaterials and heterostructures. Chem. Soc. Rev. 47, 6046–6072 (2018). 29564440 10.1039/C7CS00886D

[7] B. Saparov, D. B. Mitzi, Organic-inorganic perovskites: Structural versatility for functional materials design. Chem. Rev. 116, 4558–4596 (2016). 27040120 10.1021/acs.chemrev.5b00715

[8] W. Paritmongkol, N. S. Dahod, A. Stollmann, N. Mao, C. Settens, S. L. Zheng, W. A. Tisdale, Synthetic variation and structural trends in layered two-dimensional alkylammonium lead halide perovskites. Chem. Mater. 31, 5592–5607 (2019).

[9] T. Hang, W. Zhang, H.-Y. Ye, R.-G. Xiong, Metal-organic complex ferroelectrics. Chem. Soc. Rev. 40, 3577–3598 (2011). 21509354 10.1039/c0cs00226g

[10] X. G. Chen, X. J. Song, Z. X. Zhang, P. F. Li, J. Z. Ge, Y. Y. Tang, J. X. Gao, W. Y. Zhang, D. W. Fu, Y. M. You, R. G. Xiong, Two-dimensional layered perovskite ferroelectric with giant piezoelectric voltage coefficient. J. Am. Chem. Soc. 142, 1077–1082 (2019). 31851495 10.1021/jacs.9b12368

[11] Y. H. Kim, Y. Zhai, H. Lu, X. Pan, C. Xiao, E. A. Gaulding, S. P. Harvey, J. J. Berry, Z. V. Vardeny, J. M. Luther, M. C. Beard, Chiral-induced spin selectivity enables a room-temperature spin light-emitting diode. Science 371, 1129–1133 (2021). 33707260 10.1126/science.abf5291

[12] W. Li, Z. Wang, F. Deschler, S. Gao, R. H. Friend, A. K. Cheetham, Chemically diverse and multifunctional hybrid organic–inorganic perovskites. Nat. Rev. Mater. 2, 16099 (2017).

[13] M. Menahem, Z. Dai, S. Aharon, R. Sharma, M. Asher, Y. Diskin-Posner, R. Korobko, A. M. Rappe, O. Yaffe, Strongly anharmonic octahedral tilting in two-dimensional hybrid halide perovskites. ACS Nano 15, 10153–10162 (2021). 34003630 10.1021/acsnano.1c02022 PMC8223479

[14] M. Z. Mayers, L. Z. Tan, D. A. Egger, A. M. Rappe, D. R. Reichman, How lattice and charge fluctuations control carrier dynamics in halide perovskites. Nano Lett. 18, 8041–8046 (2018). 30387614 10.1021/acs.nanolett.8b04276

[15] D. A. Egger, A. Bera, D. Cahen, G. Hodes, T. Kirchartz, L. Kronik, R. Lovrincic, A. M. Rappe, D. R. Reichman, O. Yaffe, What remains unexplained about the properties of halide perovskites? Adv. Mater. 30, 1800691 (2018). 10.1002/adma.201800691 29569287

[16] K. Miyata, X.-Y. Zhu, Ferroelectric large polarons. Nat. Mater. 17, 379–381 (2018). 29686246 10.1038/s41563-018-0068-7

[17] J. P. Rivett, L. Z. Tan, M. B. Price, S. A. Bourelle, N. J. Davis, J. Xiao, Y. Zou, R. Middleton, B. Sun, A. M. Rappe, D. Credgington, F. Deschler, Long-lived polarization memory in the electronic states of lead-halide perovskites from local structural dynamics. Nat. Commun. 9, 3531 (2018). 30166536 10.1038/s41467-018-06009-3 PMC6117347

[18] D. Spirito, Y. Asensio, L. E. Hueso, B. Martín-García, Raman spectroscopy in layered hybrid organic-inorganic metal halide perovskites. J. Phys. Mater. 5, 034004 (2022).

[19] B. Dhanabalan, Y. C. Leng, G. Biffi, M. L. Lin, P. H. Tan, I. Infante, L. Manna, M. P. Arciniegas, R. Krahne, Directional anisotropy of the vibrational modes in 2D-layered perovskites. ACS Nano 14, 4689–4697 (2020). 32275388 10.1021/acsnano.0c00435 PMC8007126

[20] J. Hlinka, T. Ostapchuk, D. Nuzhnyy, J. Petzelt, P. Kuzel, C. Kadlec, P. Vanek, I. Ponomareva, L. Bellaiche, Coexistence of the phonon and relaxation soft modes in the terahertz dielectric response of tetragonal BaTiO3. Phys. Rev. Lett. 101, 167402 (2008). 18999713 10.1103/PhysRevLett.101.167402

[21] O. Yaffe, Y. Guo, L. Z. Tan, D. A. Egger, T. Hull, C. C. Stoumpos, F. Zheng, T. F. Heinz, L. Kronik, M. G. Kanatzidis, J. S. Owen, A. M. Rappe, M. A. Pimenta, L. E. Brus, Local polar fluctuations in lead halide perovskite crystals. Phys. Rev. Lett. 118, 136001 (2017). 28409968 10.1103/PhysRevLett.118.136001

[22] N. S. Dahod, A. France-Lanord, W. Paritmongkol, J. C. Grossman, W. A. Tisdale, Low-frequency Raman spectrum of 2D layered perovskites: Local atomistic motion or superlattice modes? J. Chem. Phys. 153, 044710 (2020). 32752687 10.1063/5.0012763

[23] O. Yaffe, A. Chernikov, Z. M. Norman, Y. Zhong, A. Velauthapillai, A. Van Der Zande, J. S. Owen, T. F. Heinz, Excitons in ultrathin organic-inorganic perovskite crystals. Phys. Rev. B 92, 045414 (2015).

[24] J. D. Ziegler, K. Q. Lin, B. Meisinger, X. Zhu, M. Kober-Czerny, P. K. Nayak, C. Vona, T. Taniguchi, K. Watanabe, C. Draxl, H. J. Snaith, J. M. Lupton, D. A. Egger, A. Chernikov, Excitons at the phase transition of 2D hybrid perovskites. ACS Photonics 9, 3609–3616 (2022).

[25] C. M. Mauck, A. France-Lanord, A. C. Hernandez Oendra, N. S. Dahod, J. C. Grossman, W. A. Tisdale, Inorganic cage motion dominates excited-state dynamics in 2D-layered perovskites (C_x_H_2x +1_NH_3_)_2_PbI_4_ (x = 4–9). J. Phys. Chem. C 123, 27904–27916 (2019).

[26] Y.-X. Yan, K. A. Nelson, Impulsive stimulated light scattering. II. Comparison to frequency-domain light-scattering spectroscopy. J. Chem. Phys. 87, 6257–6265 (1987).

[27] T. P. Dougherty, G. P. Wiederrecht, K. A. Nelson, M. H. Garrett, H. P. Jensen, C. Warde, Femtosecond resolution of soft mode dynamics in structural phase transitions. Science 258, 770–774 (1992). 17777028 10.1126/science.258.5083.770

[28] Q. Zhong, J. T. Fourkas, Optical Kerr effect spectroscopy of simple liquids. J. Phys. Chem. B. 112, 15529–15539 (2008). 19367867 10.1021/jp807730u

[29] K. Miyata, D. Meggiolaro, M. T. Trinh, P. P. Joshi, E. Mosconi, S. C. Jones, F. De Angelis, X. Y. Zhu, Large polarons in lead halide perovskites. Sci. Adv. 3, e1701217 (2017). 28819647 10.1126/sciadv.1701217 PMC5553817

[30] H. Zhu, K. Miyata, Y. Fu, J. Wang, P. P. Joshi, D. Niesner, K. W. Williams, S. Jin, X. Y. Zhu, Screening in crystalline liquids protects energetic carriers in hybrid perovskites. Science 353, 1409–1413 (2016). 27708033 10.1126/science.aaf9570

[31] S. F. Maehrlein, P. P. Joshi, L. Huber, F. Wang, M. Cherasse, Y. Liu, D. M. Juraschek, E. Mosconi, D. Meggiolaro, F. De Angelis, X.-Y. Zhu, Decoding ultrafast polarization responses in lead halide perovskites by the two-dimensional optical Kerr effect. Proc. Natl. Acad. Sci. 118, e2022268118 (2021). 33558241 10.1073/pnas.2022268118 PMC7896285

[32] F. Thouin, D. A. Valverde-Chávez, C. Quarti, D. Cortecchia, I. Bargigia, D. Beljonne, A. Petrozza, C. Silva, A. R. Srimath Kandada, Phonon coherences reveal the polaronic character of excitons in two-dimensional lead halide perovskites. Nat. Mater. 18, 349–356 (2019). 30643234 10.1038/s41563-018-0262-7

[33] P. Guo, Y. Xia, J. Gong, D. H. Cao, X. Li, X. Li, Q. Zhang, C. C. Stoumpos, M. S. Kirschner, H. Wen, V. B. Prakapenka, J. B. Ketterson, A. B. F. Martinson, T. Xu, M. G. Kanatzidis, M. K. Y. Chan, R. D. Schaller, Direct observation of bandgap oscillations induced by optical phonons in hybrid lead iodide perovskites. Adv. Funct. Mater. 30, 1907982 (2020).

[34] L. N. Quan, Y. Park, P. Guo, M. Gao, J. Jin, J. Huang, J. K. Copper, A. Schwartzberg, R. Schaller, D. T. Limmer, P. Yang, Vibrational relaxation dynamics in layered perovskite quantum wells. Proc. Natl. Acad. Sci. U.S.A. 118, e2104425118 (2021). 34131083 10.1073/pnas.2104425118 PMC8237612

[35] J. Fu, M. Li, A. Solanki, Q. Xu, Y. Lekina, S. Ramesh, Z. X. Shen, T. C. Sum, Electronic states modulation by coherent optical phonons in 2D halide perovskites. Adv. Mater. 33, 2006233 (2021). 10.1002/adma.202006233 33576093

[36] M. C. Hoffmann, N. C. Brandt, H. Y. Hwang, K.-L. Yeh, K. A. Nelson, Terahertz Kerr effect. Appl. Phys. Lett. 95, 231105 (2009).

[37] A. A. Melnikov, V. E. Anikeeva, O. I. Semenova, S. V. Chekalin, Terahertz Kerr effect in a methylammonium lead bromide perovskite crystal. Phys. Rev. B 105, 174304 (2022).

[38] L. Huber, S. F. Maehrlein, F. Wang, Y. Liu, X. Y. Zhu, The ultrafast Kerr effect in anisotropic and dispersive media. J. Chem. Phys. 154, 094202 (2021). 33685130 10.1063/5.0037142

[39] M. Frenzel, M. Cherasse, J. M. Urban, F. Wang, B. Xiang, L. Nest, L. Huber, L. Perfetti, M. Wolf, T. Kampfrath, X. Y. Zhu, S. F. Maehrlein, Nonlinear THz control of the lead halide perovskite lattice. arXiv:2301.03508 [cond-mat.mtrl-sci] (2023). 10.1126/sciadv.adg3856 PMC10208573 37224256

[40] H. Elgabarty, T. Kampfrath, D. J. Bonthuis, V. Balos, N. K. Kaliannan, P. Loche, R. R. Netz, M. Wolf, T. D. Kuhne, M. Sajadi, Energy transfer within the hydrogen bonding network of water following resonant terahertz excitation. Sci. Adv. 6, eaay7074 (2020). 32494631 10.1126/sciadv.aay7074 PMC7182424

[41] H. Zhao, Y. Tan, L. Zhang, R. Zhang, M. Shalaby, C. Zhang, Y. Zhao, X. Zhang, Ultrafast hydrogen bond dynamics of liquid water revealed by terahertz-induced transient birefringence. Light Sci. Appl. 9, 136 (2020). 32802323 10.1038/s41377-020-00370-z PMC7403349

[42] G. Khalsa, N. A. Benedek, J. Moses, Ultrafast control of material optical properties via the infrared resonant Raman effect. Phys. Rev. X 11, 021067 (2021).

[43] M. Först, C. Manzoni, S. Kaiser, Y. Tomioka, Y. Tokura, R. Merlin, A. Cavalleri, Nonlinear phononics as an ultrafast route to lattice control. Nat. Phys. 7, 854–856 (2011).

[44] D. M. Juraschek, S. F. Maehrlein, Sum-frequency ionic Raman scattering. Phys. Rev. B 97, 174302 (2018).

[45] C. Aku-Leh, J. Zhao, R. Merlin, J. Menendez, M. Cardona, Long-lived optical phonons in ZnO studied with impulsive stimulated Raman scattering. Phys. Rev. B 71, 205211 (2005).

[46] C. L. Johnson, B. E. Knighton, J. A. Johnson, Distinguishing nonlinear terahertz excitation pathways with two-dimensional spectroscopy. Phys. Rev. Lett. 122, 073901 (2019). 30848646 10.1103/PhysRevLett.122.073901

[47] S. Maehrlein, A. Paarmann, M. Wolf, T. Kampfrath, Terahertz sum-frequency excitation of a Raman-active phonon. Phys. Rev. Lett. 119, 127402 (2017). 29341630 10.1103/PhysRevLett.119.127402

[48] Y.-X. Yan, E. B. Gamble Jr, K. A. Nelson, Impulsive stimulated scattering: General importance in femtosecond laser pulse interactions with matter, and spectroscopic applications. J. Chem. Phys. 83, 5391–5399 (1985).

[49] A. Maradudin, R. Wallis, Ionic Raman effect. I. Scattering by localized vibration modes. Phys. Rev. B 2, 4294–4299 (1970).

[50] R. Wallis, A. Maradudin, Ionic Raman effect. II. The first-order ionic Raman effect. Phys. Rev. B 3, 2063 (1971).

[51] L. Humphreys, Ionic raman effect. III. First- and second-order ionic Raman effects. Phys. Rev. B 6, 3886–3897 (1972).

[52] X. Li, T. Qiu, J. Zhang, E. Baldini, J. Lu, A. M. Rappe, K. A. Nelson, Terahertz field–induced ferroelectricity in quantum paraelectric SrTiO_3_. Science 364, 1079–1082 (2019). 31197011 10.1126/science.aaw4913

[53] A. von Hoegen, M. Fechner, M. Forst, N. Taherian, E. Rowe, A. Ribak, J. Porras, B. Keimer, M. Michael, E. Demler, A. Cavalleri, Amplification of superconducting fluctuations in driven YBa_2_Cu_3_O_6+x_. Phys. Rev. X 12, 031008 (2022).

[54] G. Mead, H.-W. Lin, I.-B. Magdau, T. F. Miller III, G. A. Blake, Sum-frequency signals in 2D-terahertz-terahertz-Raman spectroscopy. J. Phys. Chem. B. 124, 8904–8908 (2020). 32897705 10.1021/acs.jpcb.0c07935

[55] M. A. Reyes-Martinez, P. Tan, A. Kakekhani, S. Banerjee, A. A. Zhumekenov, W. Peng, O. M. Bakr, A. M. Rappe, Y.-L. Loo, Unraveling the elastic properties of (quasi) two-dimensional hybrid perovskites: A joint experimental and theoretical study. ACS Appl. Mater. Interfaces 12, 17881–17892 (2020). 32188240 10.1021/acsami.0c02327

[56] X. Gonze, Adiabatic density-functional perturbation theory. Phys. Rev. A 52, 1096–1114 (1995). 9912349 10.1103/physreva.52.1096

[57] S. Baroni, P. Giannozzi, A. Testa, Green’s-function approach to linear response in solids. Phys. Rev. Lett. 58, 1861 (1987), 1864. 10034557 10.1103/PhysRevLett.58.1861

[58] S. Baroni, S. De Gironcoli, A. Dal Corso, P. Giannozzi, Phonons and related crystal properties from density-functional perturbation theory. Rev. Mod. Phys. 73, 515 (2001), 562.

[59] A. S. Disa, T. F. Nova, A. Cavalleri, Engineering crystal structures with light. Nat. Phys. 17, 1087–1092 (2021).

[60] G. Grinblat, I. Abdelwahab, M. P. Nielsen, P. Dichtl, K. Leng, R. F. Oulton, K. P. Loh, S. A. Maier, Ultrafast all-optical modulation in 2D hybrid perovskites. ACS Nano 13, 9504–9510 (2019). 31314482 10.1021/acsnano.9b04483

[61] Z. Sun, A. Martinez, F. Wang, Optical modulators with 2D layered materials. Nat. Photonics 10, 227–238 (2016).

[62] C. C. Stoumpos, D. H. Cao, D. J. Clark, J. Young, J. M. Rondinelli, J. I. Jang, J. T. Hupp, M. G. Kanatzidis, Ruddlesden–Popper hybrid lead iodide perovskite 2D homologous semiconductors. Chem. Mater. 28, 2852–2867 (2016).

[63] K.-L. Yeh, J. Hebling, M. C. Hoffmann, K. A. Nelson, Generation of high average power 1 kHz shaped THz pulses via optical rectification. Opt. Commun. 281, 3567–3570 (2008).

[64] F. Y. Gao, Z. Zhang, Z.-J. Liu, K. A. Nelson, High-speed two-dimensional terahertz spectroscopy with echelon-based shot-to-shot balanced detection. Opt. Lett. 47, 3479–3482 (2022). 35838708 10.1364/OL.462624

[65] J. P. Perdew, K. Burke, M. Ernzerhof, Generalized gradient approximation made simple. Phys. Rev. Lett. 77, 3865–3868 (1996). 10062328 10.1103/PhysRevLett.77.3865

[66] A. M. Rappe, K. M. Rabe, E. Kaxiras, J. D. Joannopoulos, Optimized pseudopotentials. Phys. Rev. B 41, 1227–1230 (1990). 10.1103/physrevb.41.1227 9993827

[67] Opium-pseudopotential generation project; http://opium.sourceforge.net.

[68] M. Thomas, M. Brehm, R. Fligg, P. Vöhringer, B. Kirchner, Computing vibrational spectra from ab initio molecular dynamics. Phys. Chem. Chem. Phys. 15, 6608–6622 (2013). 23416970 10.1039/c3cp44302g

[69] R. Sharma, Z. Dai, L. Gao, T. M. Brenner, L. Yadgarov, J. Zhang, Y. Rakita, R. Korobko, A. M. Rappe, O. Yaffe, Elucidating the atomistic origin of anharmonicity in tetragonal CH_3_NH_3_PbI_3_ with Raman scattering. Phys. Rev. Mater. 4, 092401 (2020).

[70] R. Sharma, M. Menahem, Z. Dai, L. Gao, T. M. Brenner, L. Yadgarov, J. Zhang, Y. Rakita, R. Korobko, I. Pinkas, A. M. Rappe, O. Yaffe, Lattice mode symmetry analysis of the orthorhombic phase of methylammonium lead iodide using polarized Raman. Phys. Rev. Mater. 4, 051601 (2020).

[71] N. Wiener, Generalized harmonic analysis. Acta Math. 55, 117–258 (1930).

[72] A. Khintchine, Korrelationstheorie der stationären stochastischen prozesse. Math. Ann. 109, 604–615 (1934).

[73] P. Giannozzi, S. Baroni, N. Bonini, M. Calandra, R. Car, C. Cavazzoni, D. Ceresoli, G. L. Chiarotti, M. Cococcioni, QUANTUM ESPRESSO: A modular and open-source software project for quantum simulations of materials. J. Phys. Condens. Matter 21, 395502 (2009). 21832390 10.1088/0953-8984/21/39/395502

[74] P. Giannozzi, O. Andreussi, T. Brumme, O. Bunau, M. B. Nardelli, M. Calandra, R. Car, C. Cavazzoni, D. Ceresoli, M. Cococcioni, Advanced capabilities for materials modelling with Quantum ESPRESSO. J. Phys. Condens. Matter 29, 465901 (2017). 29064822 10.1088/1361-648X/aa8f79

[75] S. Nosé, A unified formulation of the constant temperature molecular dynamics methods. J. Chem. Phys. 81, 511–519 (1984).

[76] W. G. Hoover, Canonical dynamics: Equilibrium phase-space distributions. Phys. Rev. A 31, 1695 (1985), 1697. 10.1103/physreva.31.1695 9895674

[77] L. Li, X. Liu, Y. Li, Z. Xu, Z. Wu, S. Han, K. Tao, M. Hong, J. Luo, Z. Sun, Two-dimensional hybrid perovskite-type ferroelectric for highly polarization-sensitive shortwave photodetection. J. Am. Chem. Soc. 141, 2623–2629 (2019). 30661350 10.1021/jacs.8b12948

[78] L. Dou, A. B. Wong, Y. Yu, M. Lai, N. Kornienko, S. W. Eaton, A. Fu, C. G. Bischak, J. Ma, T. Ding, N. S. Ginsberg, L. Wang, A. P. Alivisatos, P. Yang, Atomically thin two-dimensional organic-inorganic hybrid perovskites. Science 349, 1518–1521 (2015). 26404831 10.1126/science.aac7660

[79] M. Hase, K. Mizoguchi, H. Harima, S.-i. Nakashima, K. Sakai, Dynamics of coherent phonons in bismuth generated by ultrashort laser pulses. Phys. Rev. B 58, 5448–5452 (1998).

[80] A. P. Thompson, H. M. Aktulga, R. Berger, D. S. Bolintineanu, W. M. Brown, P. S. Crozier, P. J. in 't Veld, A. Kohlmeyer, S. G. Moore, T. D. Nguyen, R. Shan, M. J. Stevens, J. Tranchida, C. Trott, S. J. Plimpton, Lammps - a flexible simulation tool for particle-based materials modeling at the atomic, meso, and continuum scales. Comput. Phys. Commun. 271, 108171 (2022).

[81] V. Kumar, M. Casella, E. Molotokaite, D. Gatti, P. Kukura, C. Manzoni, D. Polli, M. Marangoni, G. Cerullo, Balanced-detection Raman-induced Kerr-effect spectroscopy. Phys. Rev. A 86, 053810 (2012).

