# Supplementary material for: Discovery of enhanced lattice dynamics in a single-layered hybrid perovskite
Source: Sci Adv. 2023 Aug 16;9(33):eadg4417. doi: 10.1126/sciadv.adg4417 (PMC10431705; doi:10.1126/sciadv.adg4417)
Supplement: Supplementary file 1 — Figs. S1 to S11 Table S1 Legends for movies S1 to S5 References [file sciadv.adg4417_sm.pdf]

Science Advances
AAAS

Supplementary Materials for

Discovery of enhanced lattice dynamics in a single-layered hybrid perovskite

Zhuquan Zhang *et al.*

Corresponding author: Edoardo Baldini, edoardo.baldini@austin.utexas.edu; Keith A. Nelson, kanelson@mit.edu

Sci. Adv. **9**, eadg4417 (2023)
DOI: 10.1126/sciadv.adg4417

The PDF file includes:

Figs. S1 to S11
Table S1
Legends for movies S1 to S5
References

Other Supplementary Material for this manuscript includes the following:

Movies S1 to S5

Supplementary Note 1: Crystal structures of bormide 2DHPs

A. X-ray diffraction

To determine the crystal structures of $(BA)_2PbBr_4$ and $(BA)_2MAPb_2Br_7$ 2DHPs, we conducted powder X-ray diffraction (PXRD) measurements on both samples. Figure. S1 displays a comparison between the experimental and simulated PXRD patterns. The experimental diffraction patterns were collected from the n = 1 and n = 2 samples, while the simulated patterns were generated using the previously reported crystal structures *(77,78)*. We found that both patterns match well with the reported results, thus confirming that $(BA)_2PbBr_4$ and $(BA)_2MAPb_2Br_7$ crystallize in the *Pbca* and $Cmc2_1$ space groups, respectively.

To investigate the temperature dependence of the crystal structures, we collected PXRD patterns of the n=1 and n=2 samples across a wide temperature range from 73 K to 298 K, as shown in Figs. S2 and S3. We found no substantial changes in the diffraction patterns within this temperature range, indicating the absence of a structural phase transition. This observation is consistent with the Raman spectra obtained over the same temperature range, as shown in Figs. 1B and 1C in the main text.

B. Polarized light imaging

We present evidence of the single crystalline nature of the crystals used in our experiments by using polarized light imaging, which allows us to observe the birefringence of

the crystal and determine its crystallographic orientation. Figures S4 A and B depict the polarized light mimeographs for the n=1 and n=2 flakes, respectively. The images reveal a uniform brightness across the entire crystal, indicating that the samples are indeed single-crystalline.

Supplementary Note 2: Comparison of TKE signals for n=1 and n=2 2DHPs

A. Dielectric properties of lead-bromide 2DHPs

In our TKE experiments, the THz-field-induced anisotropic responses are detected by the depolarization of the probe pulse at 1.55 eV. This photon energy is much lower than the bandgaps of both n=1 and n=2 2DHPs, and therefore their dielectric properties at 1.55 eV are very similar. To clarify this, we show the optical absorption and photoluminescence spectra adapted from Ref. *(8)* in Figure S5. For the n=1 samples, the lowest excitonic resonance lies at around 3.10 eV and this value is shifted to $\sim$ 2.76 eV for n=2. This set of data demonstrate that our probe photon energy is indeed well below the optical gaps and the TKE signals are not affected by the difference in the dielectric properties of both samples.

B. TKE response of n-butylammonium bromide

To provide additional clues about the origins of the initial bipolar responses in the TKE signals from the n=1 sample, we also performed TKE experiments on crystalline n-

butylammonium bromide (98%, SIGMA-ALDRICH) at room temperature. As shown in Figure. S6, the TKE signal from n-butylammonium bromide also displays a strong bipolar response, similar to what is observed in the n=1 2DHP. Consequently, it appears plausible that, alongside the attribution to the nonlinear propagation effect, the observed bipolar response in the n=1 2DHP could be influenced by the presence of the n-butylammonium (BA) spacers. However, this bipolar response is not observed in the n=2 2DHP, which has an additional lead bromide octahedral layer and more crucially, the methylammonium organic cations. Therefore, it is likely that this more complicated structure of the n=2 system leads to the suppression of the bipolar response.

Supplementary Note 3: Sum-frequency excitation of the Raman mode

In this section, we provide a comparison of the two nonlinear excitation pathways to drive the Raman mode. For a two-photon Raman scattering process, we assume a harmonic lattice potential $V(Q_R) = \frac{1}{2}\Omega_R^2 Q_R^2$, and the corresponding Lagrange equation with a classical Raman type harmonic-oscillator can be derived as *(47,48)*

$$(\frac{\partial^2}{\partial t^2} + \Gamma_R \frac{\partial}{\partial t} + \Omega_R^2) Q_R = E(t)^2 \frac{\partial \chi}{\partial Q_R}. \tag{1}$$

Here, Q_R represents the normal Raman mode coordinate, Γ_R is a phenomenological damping term, Ω_R is the eigenfrequency of the Raman mode, $E(t)$ represents the driving

electric field, and χ is the linear dielectric susceptibility. The excitation of the Raman mode is mediated by the Raman tensor $\frac{\partial \chi}{\partial Q_R}$. Since the driving term on the right-hand side of the equation scales as the square of the pump electric field, which can couple to the Raman mode through either difference- (i.e., $\Omega_1 - \Omega_2 = \Omega_R$) or sum-frequency (i.e., $\Omega_1 + \Omega_2 = \Omega_R$) components of light, this equation of motion describes impulsive stimulated Raman scattering as well as sum-frequency excitation observed here. It is worth noting that the dielectric responses of hybrid perovskites feature large jumps in the THz range as the frequency decreases across several broad transverse optical phonon resonances. Since for both Raman excitation processes, the pump electric field interacts with virtual electronic dipole transitions, THz off-resonance excitation gives rise to colossal nonlinear polarizability response compared to that in the optical range. This can be also viewed in the time domain as a cloud of electrons bound to a nucleus displaces more strongly in response to a slowly varying electric field as compared to the ultrafast optical pump pulses. The non-impulsive nature of the THz sum-frequency excitation mechanism may also explain why the THz pump pulse selectively drives the octahedral twist mode with the largest polarizability. The quasi-DC electric field continuously builds up the phonon amplitude, whereas in the optical impulsive stimulated Raman scattering process, all Raman phonons within the pump bandwidth are coherently excited *(34)*.

In contrast, the ionic Raman scattering requires an anharmonic lattice potential and its simplest form can be described as $V(Q_R) = \frac{1}{2}\Omega_R^2 Q_R^2 + \frac{1}{2}\Omega_{IR}^2 Q_{IR}^2 + cQ_{IR}Q_R^2$,

where c is the anharmonic coupling coefficient. The corresponding equations of motion are *(43)*

$$(\frac{\partial^2}{\partial t^2} + \Gamma_{IR}\frac{\partial}{\partial t} + \Omega_{IR}^2 + 2cQ_R)Q_{IR} = Z_{IR}E(t), \tag{2}$$

$$(\frac{\partial^2}{\partial t^2} + \Gamma_R\frac{\partial}{\partial t} + \Omega_R^2)Q_R = cQ_{IR}(t)^2, \tag{3}$$

where in the first equation Z_{IR} is the effective charge of the infrared-active phonon mode. In this case, the Raman mode is activated by anharmonic coupling to the directly driven infrared-active mode. For this process to be efficient, there should exist an infrared-active phonon mode with its eigenfrequency that matches the sum-frequency excitation condition ($\Omega_{IR} = \frac{1}{2}\Omega_R$, i.e., $\sim$ 0.9 THz), which is ruled out by the time-domain THz spectroscopy measurement. Therefore, we confirm that the driven Raman mode excited through large polarizability rather than anharmonicity.

Supplementary Note 4: Decay process of the driven Raman mode

In this section, we provide additional details on the fitting and interpretation of the temperature-dependent decay rates of the driven Raman mode in the n=1 sample. As shown in Figure 2D, a notable reduction in the decay rate is observed with decreasing temperature. The raw data, along with the fits, are presented in Figure. S7.

In general, the coherent phonon decay process involves a combination of anhar-

monic decay, which occurs via anharmonic coupling to acoustic phonons, and pure dephasing. To obtain a more comprehensive understanding of the temperature-dependent decay rates, we fit the data to the following equation:

$$\Gamma_R = \Gamma_0[1 + \frac{2}{exp(\frac{\hbar\Omega_R}{2k_BT}) - 1}] + \Gamma_1, \qquad (4)$$

where the first term represents the temperature-dependent anharmonic decay rate *(79)*. Here, Γ_0 is the effective anharmonic decay constant, Ω_R is the frequency of the Raman mode, and k_B is the Boltzmann constant. The second term corresponds to the temperature-independent phonon decay rate, such as via phonon-defect scattering processes. The fits agree well with the experimental data, with Γ_0 and Γ_1 determined to be 0.026 ps^{-1} and 0.059 ps^{-1}, respectively. These results suggest that the decay of the Raman coherence is dominated by the anharmonic decay channel at high temperatures. Previous studies have shown that 2DHPs feature optically-inactive acoustic branches as well as zone-folded longitudinal acoustic phonons inherent to the alternating layers *(22)*, which may also contribute to the efficient decay of the Raman coherence.

Note that our findings appear to contradict a previous study that employed transient absorption spectroscopy with impulsive stimulated Raman scattering *(34)*. This earlier investigation did not observe any long-lived phonon coherence in $(BA)_2PbBr_4$ and concluded that a temperature-independent decay rate existed based on this observa-

tion. However, our study, which used THz sum-frequency excitation, reveals long-lived Raman coherence of the octahedral twist mode with a temperature-dependent decay rate. First, in Ref. *(34)*, the impulsive stimulated Raman scattering mechanism excites all the Raman resonances within the bandwidth of the pump pulse, and the white light probe is only sensitive to the Raman mode that couples to the excited electronic state. In our case, the THz sum-frequency excitation is non-impulsive in nature and selectively drives the most polarizable Raman mode at 1.8 THz; moreover, the Kerr detection probe is sensitive to any driven Raman mode, regardless of whether it couples to the excited state. This difference in experimental technique likely explains why such a long-lived Raman coherence was not observed in the previous study using impulsive stimulated Raman scattering and highlights the strength of using intense THz pulses to coherently drive low-energy collective modes in hybrid perovskites. As for the MD simulation, Ref. *(34)* only considered the lowest frequency optical phonon mode, which may couple to the organic ligands more efficiently than the octahedral twist mode discussed here.

Supplementary Note 5: MD simulation details

A. Finite temperature calculations

We used a Nosé-Hoover thermostat reference (adapted from LAMMPS) to sample the thermodynamics of the system, where the thermal damping time and targeted temperature were set at 1 ps and 77 K, respectively. *(80)* To ensure a high-frequency resolution,

we calculated the auto-correlation function using 600 steps, yielding a frequency resolution of approximately 0.017 THz, which is much narrower than any Raman responses of interest. Figure. S8 shows the time evolution of the non-zero tensor elements of the dielectric susceptibility in thermal equilibrium along with the temperature fluctuations (See inset plots). The spontaneous Raman scattering intensity was calculated based on the isotropic average condition *(68)*,

$$I_{//} \propto \frac{(\omega_{in} - \omega_p)^4}{\omega_p} \frac{45a_p^2 + 4\gamma_p^2}{45} \frac{1}{1 - exp(-\frac{\hbar\omega_p}{k_B T})}, \tag{5}$$

$$I_{\perp} \propto \frac{(\omega_{in} - \omega_p)^4}{\omega_p} \frac{3\gamma_p^2}{45} \frac{1}{1 - exp(-\frac{\hbar\omega_p}{k_B T})}, \tag{6}$$

where $I_{//}$ and $I_{\perp}$ denote the Raman scattering intensity polarized parallel and perpendicular to the incident light polarization; ω_{in}, ω_p are the frequencies of the incident and scattered light; and Q$_p$ represents the normal mode coordinate; a_p is the isotropic polarizability, defined as

$$a_p = \frac{1}{3}(\frac{\partial\chi_{xx}}{\partial Q_p} + \frac{\partial\chi_{yy}}{\partial Q_p} + \frac{\partial\chi_{zz}}{\partial Q_p}), \tag{7}$$

and γ_p is the anisotropic polarizability, defined as

$$\gamma_p^2 = \frac{1}{2}(\frac{\partial\chi_{xx}}{\partial Q_p} - \frac{\partial\chi_{yy}}{\partial Q_p})^2 + \frac{1}{2}(\frac{\partial\chi_{yy}}{\partial Q_p} - \frac{\partial\chi_{zz}}{\partial Q_p})^2 + \frac{1}{2}(\frac{\partial\chi_{zz}}{\partial Q_p} - \frac{\partial\chi_{xx}}{\partial Q_p})^2 + 3(\frac{\partial\chi_{xy}}{\partial Q_p})^2 + 3(\frac{\partial\chi_{yz}}{\partial Q_p})^2 + 3(\frac{\partial\chi_{xz}}{\partial Q_p})^2. \quad (8)$$

The Raman tensor $\frac{\partial\chi_{ij}}{\partial Q_p}$ was calculated by computing the time-domain auto-correlation function

$$(\frac{\partial\chi_{ij}}{\partial Q_p})^2 \propto \int < \chi_{ij}(\tau)\chi_{ij}(t+\tau) >_\tau e^{-iw_p t}dt. \quad (9)$$

We also conducted further tests to evaluate the influence of thermostat parameters, specifically adjusting the damping time to 0.1 ps. As depicted in Fig. S9, the recalculated Raman spectra for both 2DHPs demonstrate qualitative and quantitative alignment with those shown in Fig. 4A of the main text. This consistency in results across different damping parameters serves to underscore the robustness of our simulations, thereby affirming that our findings are indeed invariant to the specific choice of damping parameter used.

B. Real-space analysis

To identify the lattice displacements corresponding to the Raman peaks, we filtered the trajectories of the system $\vec{X}(t)$ with each peak frequency Ω_R and a window $\Delta\omega$ to get

the real-space trajectory with the mode frequency equals to Ω_R:

$$\vec{X}'(t, \Omega_R) = \mathcal{FT}^{-1}\{\Theta(\omega - \Omega_R + \Delta\omega)\Theta(\omega - \Omega_R - \Delta\omega)\mathcal{FT}\{\vec{X}(t)\}\} \tag{10}$$

where Θ is the Heaviside step function, and $\mathcal{FT}$ denotes the Fourier transform. We attached supplementary video files for each distinct lattice motion, corresponding to Raman peaks at 0.7 THz, 1.1 THz, 1.5 THz and 1.8 THz frequencies. The 0.7 THz mode coincides the shearing motion of the two adjacent octahedral layers. The 1.1 THz mode represents the breathing motion of the octahedral layers. The 1.5 THz and 1.8 THz modes correspond to the octahedral bending and twisting motions.

C. Simulation of organic cation ordering

To evaluate the impact of molecular moieties on the dynamical disorder, we compute the auto-correlation function of the ordering of each organic ligand chain for both the n=1 and n=2 systems. Specifically, we define the auto-correlation function as follows:

$$C(t) = \langle \vec{d}(\tau - t) \cdot \vec{d}(\tau) \rangle_\tau \tag{11}$$

Here, $\vec{d}$ represents a vector indicating the direction of each organic cation from head to tail. Figure S10 depicts the auto-correlation functions of six individual BA cations in the n=1 2DHP and four BA cations along with two MA cations in the n=2 2DHP. The auto-correlation functions of all BA cations in both systems exhibit some oscillatory

responses with small amplitude modulations (i.e., $\sim 1\%$). These results suggest that the BA chains only oscillate gently and do not reorder at finite temperatures, which is consistent with the absence of any structural phase transition. In stark contrast, the auto-correlation functions of the two MA cations in the n=2 2DHP show much lower auto-correlation values, indicative of local and stochastic motion. Such spatially uncorrelated dynamics lead to the broadening of the steady-state Raman response in the n=2 2DHP and explain why long-lived Raman coherence is only observed in the n=1 system.

We then investigate the time-dependent reorientation dynamics of MA molecules during finite temperature MD simulation, as shown in Figure S11A. We find that for n=1 2DHP has C-N bond homogenous local reorientation around the Pb-Br octahedral, while n=2 2DHP has inhomogenous local reorientation. Such local inhomogeneity came from the chemical environment difference. In n=1 2DHP, all the C-N bonds are having the same chemical environments, thus their oscillations are nearly the same. However, for n=2 2DHP, their oscillations are inequivalent. Such inhomogenous response broad the lineshape. The spherical coordinates are build based on the C as origin and normal to the octahedral normal direction as pole. The spectrum of oscillation is defined as:

$$\theta(w) = \mathcal{FT}\{\theta(t)\} \tag{12}$$

$$\phi(w) = \mathcal{FT}\{\phi(t)\} \tag{13}$$

Supplementary Note 6: Evaluation of Kerr constant and refractive modulation depth

In this section, we provide an estimate of the Kerr nonlinear coefficient and the refractive modulation depth of the n=1 2DHP under the intense THz fields. When irradiating the material with a peak THz electric field strength of 610 kV/cm at room temperature, we observe a 1.2$\%$ deviation from the balanced signals at the arrival of the THz peak. In the balanced detection scheme using a half-wave plate *(81)*, the differential signal is

$$\frac{\Delta I}{I_0} = \frac{I_1 - I_2}{I_1 + I_2} = \frac{1}{2} sin(2\Gamma), \tag{14}$$

where I_1 and I_2 are intensities of the two orthogonally polarized beams measured by a pair of identical photodiodes. The phase shift is $\Gamma = 2\pi \Delta n L / \lambda_{800nm}$, with L being the sample thickness (i.e., $\sim$ 100μm) and Δn being the THz-induced change in refractive index. For small polarization rotations, $\Delta I / I_0 \sim 2\pi \Delta n L / \lambda_{800nm}$, from which we calculate $\Delta n = 1.528 \times 10^{-5}$, and the Kerr constant $K = \Delta n / (\lambda_{800nm} E_{THz}^2) = 5.132 \times 10^{-15} m \cdot V^{-2}$. The modulation amplitude is calculated as $10 \times log((I_0 + \Delta I)/(I_0 + \Delta I)) = 0.24$ dB, which means the modulation depth is approximately 2.4 dB/mm. This suggests that 2DHPs are promising candidates for achieving high-speed, all-optical, and broadband refractive modulators.

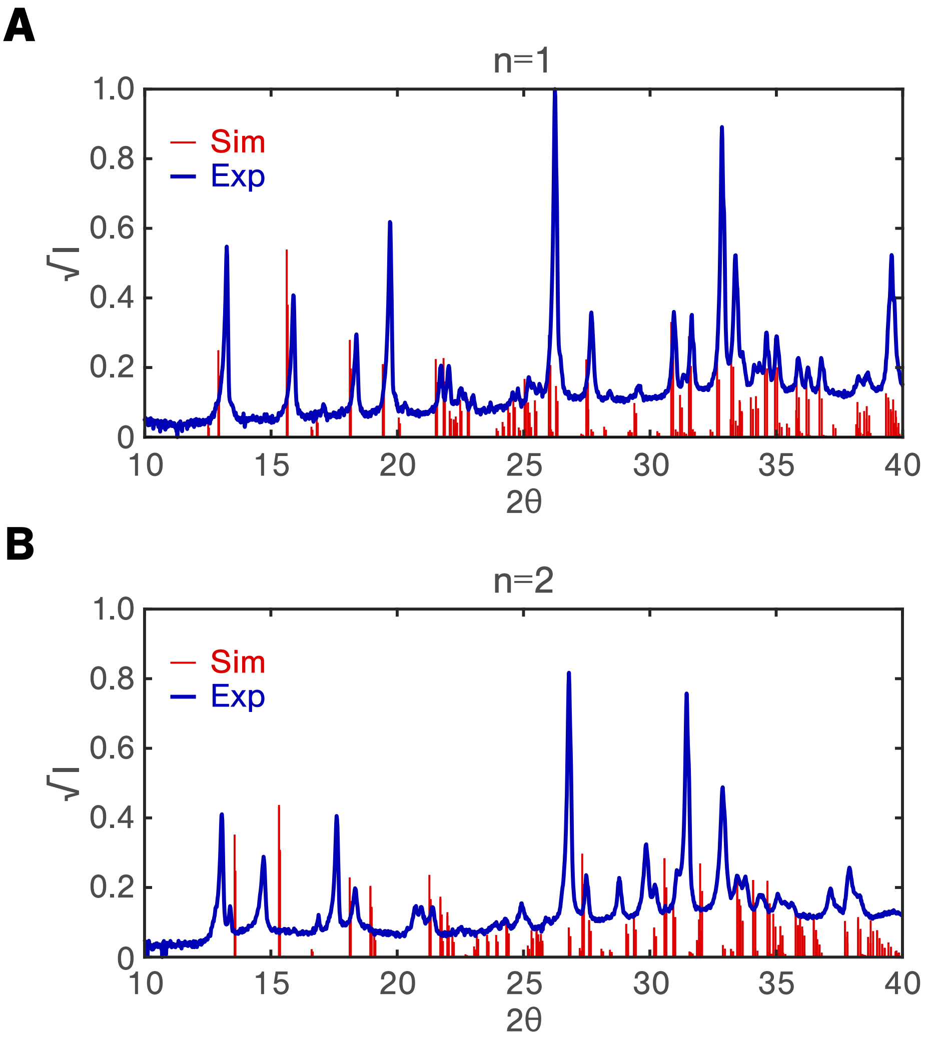

Fig. S1: Comparison between experimental and simulated powder X-ray diffraction patterns of $(BA)_2PbBr_4$ and $(BA)_2MAPb_2Br_7$ samples. (**A**) Experimental data for n=1 at room temperature and simulated pattern calculated based on the previously reported structure at 100 K. (**B**) Experimental data for n=2 at room temperature and simulated pattern calculated based on the previously reported structure at 200 K. The slight shift observed between the simulated and experimental patterns indicates a slight mismatch between the lattice constants, but the structural space group $Cmc2_1$ has been confirmed.

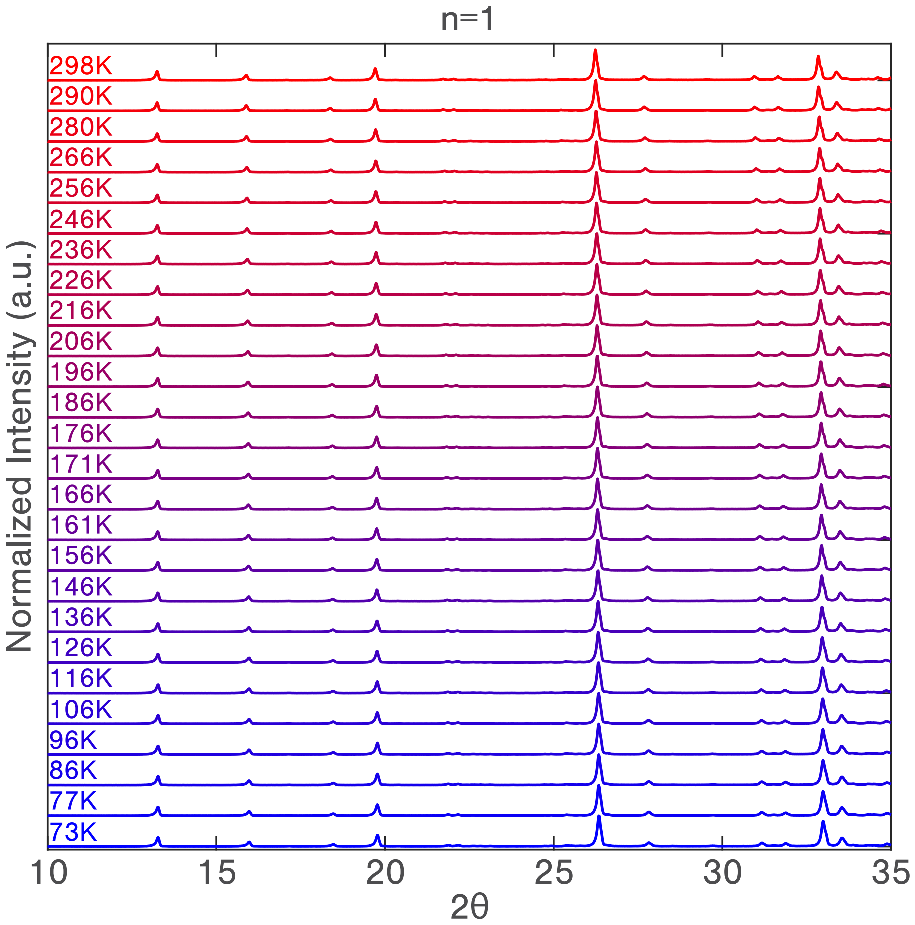

Fig. S2: Temperature-dependent powder X-ray diffraction patterns of $(BA)_2PbBr_4$ from 73 K to 298 K.

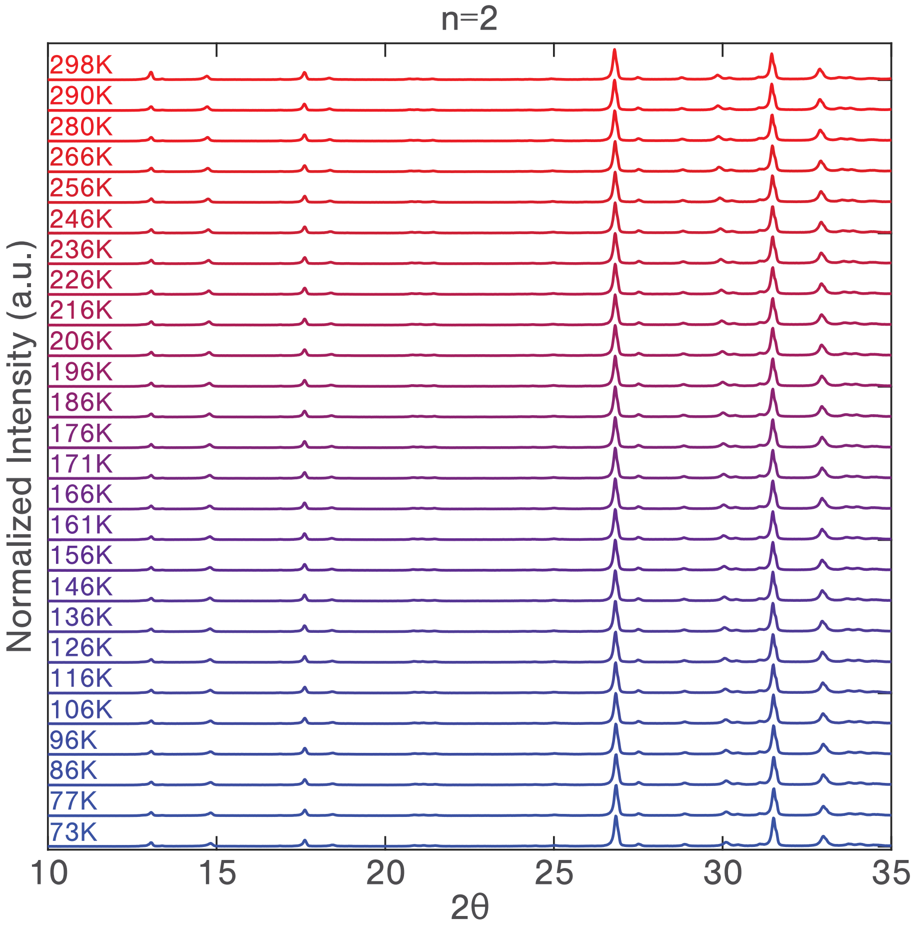

Fig. S3: Temperature-dependent powder X-ray diffraction patterns of $(BA)_2MAPb_2Br_7$ from 73 K to 298 K.

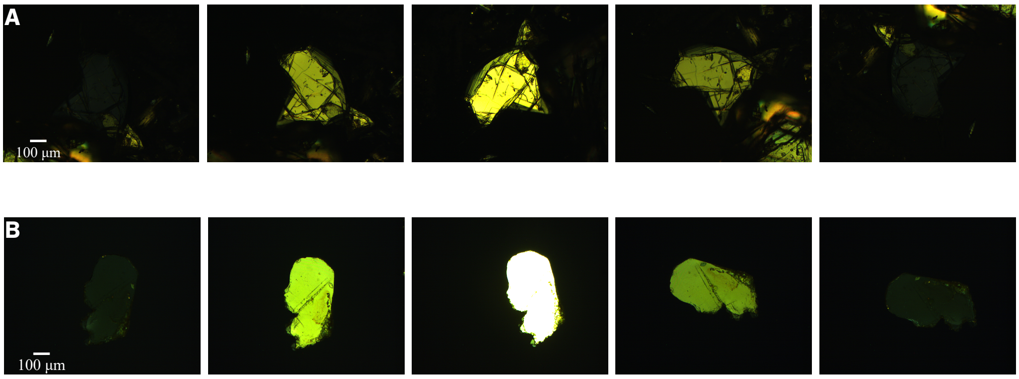

Fig. S4: Polarized light imaging measurements. Polarized optical micrographs of representative n=1 (**A**) and n=2 (**B**) flakes rotated at different angles with respect to the incident light polarization. The uniform brightness throughout the crystals at each orientation suggests that the synthesized crystals are single-crystalline.

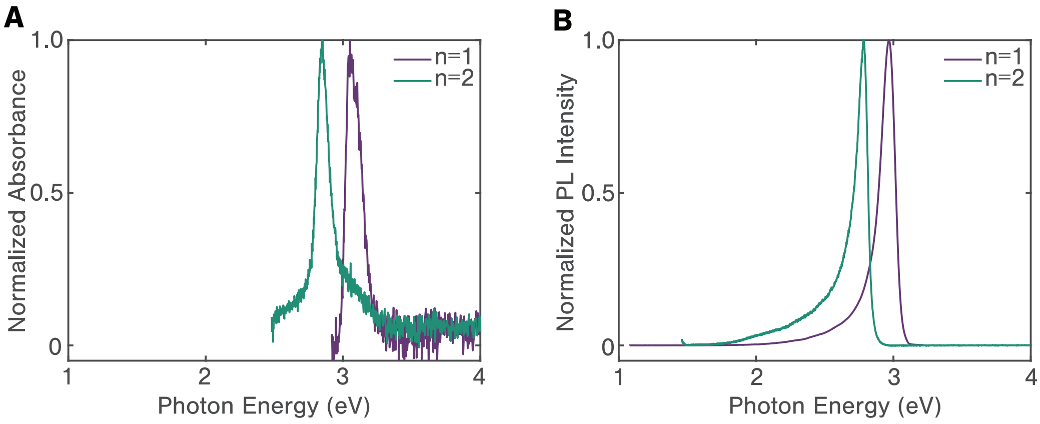

Fig. S5: Optical properties of 2DHPs. Absorption (**A**) and photoluminescence (**B**) spectra of n=1 and n=2 2DHPs adapted from Ref [4].

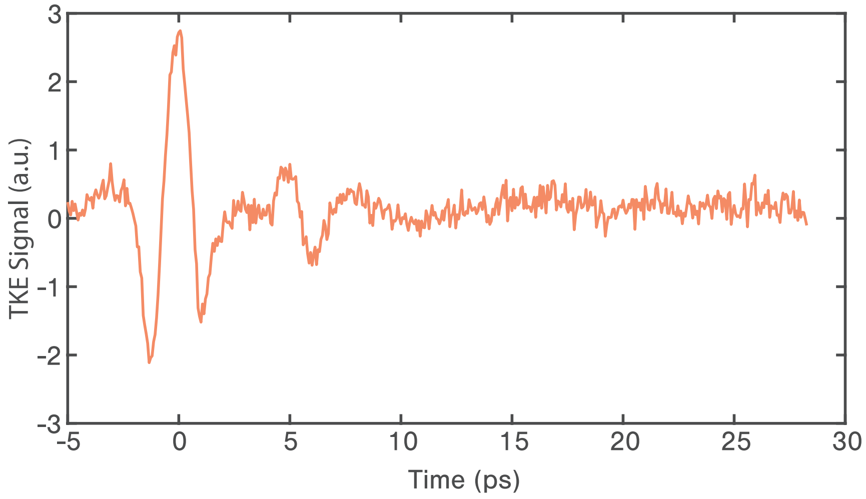

Fig. S6: TKE signals of n-butylammonium bromide crystal at room temperature. The initial TKE signal shows a bipolar response similar to that observed in the n=1 sample. The second peak at ~ 5 ps is due to the reflection of the THz pulse within crystal.

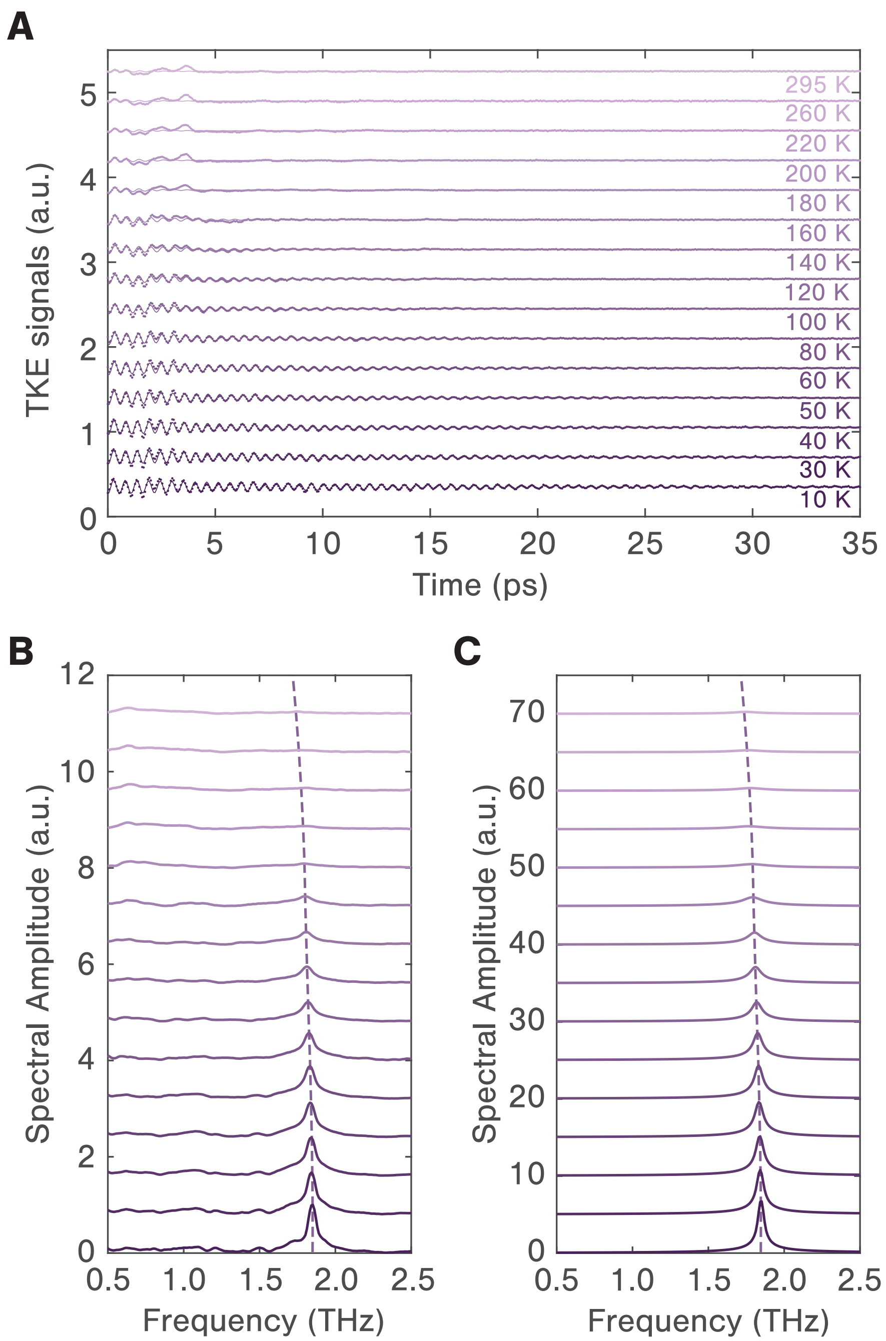

Fig. S7: Temperature-dependent TKE signals of n=1 2DHP. (**A**) Raw data (dots) and fits (solid lines) to the Raman coherence signals as a function of temperature. (**B**) Fourier transform of the raw data. (**C**) Fourier transform of the fits. The dashed curves in (B) and (C) are guides to the eye showing the frequency of the Raman mode.

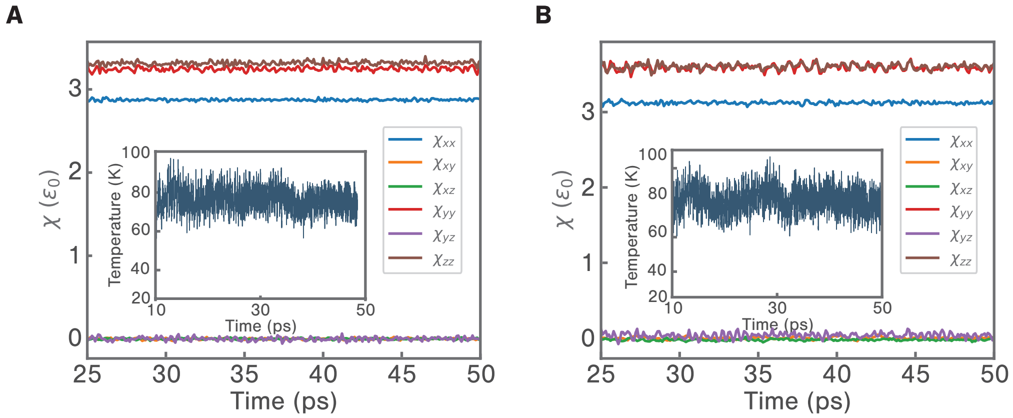

Fig. S8: Time evolution of susceptibilities of 2DHPs at 77 K. Non-zero tensor components of n=1 (**A**) and n=2 (**B**) of time-dependent susceptibilities sampled from finite-temperature trajectories at every 100 fs. Inset plots show the corresponding thermal fluctuations during the same time interval.

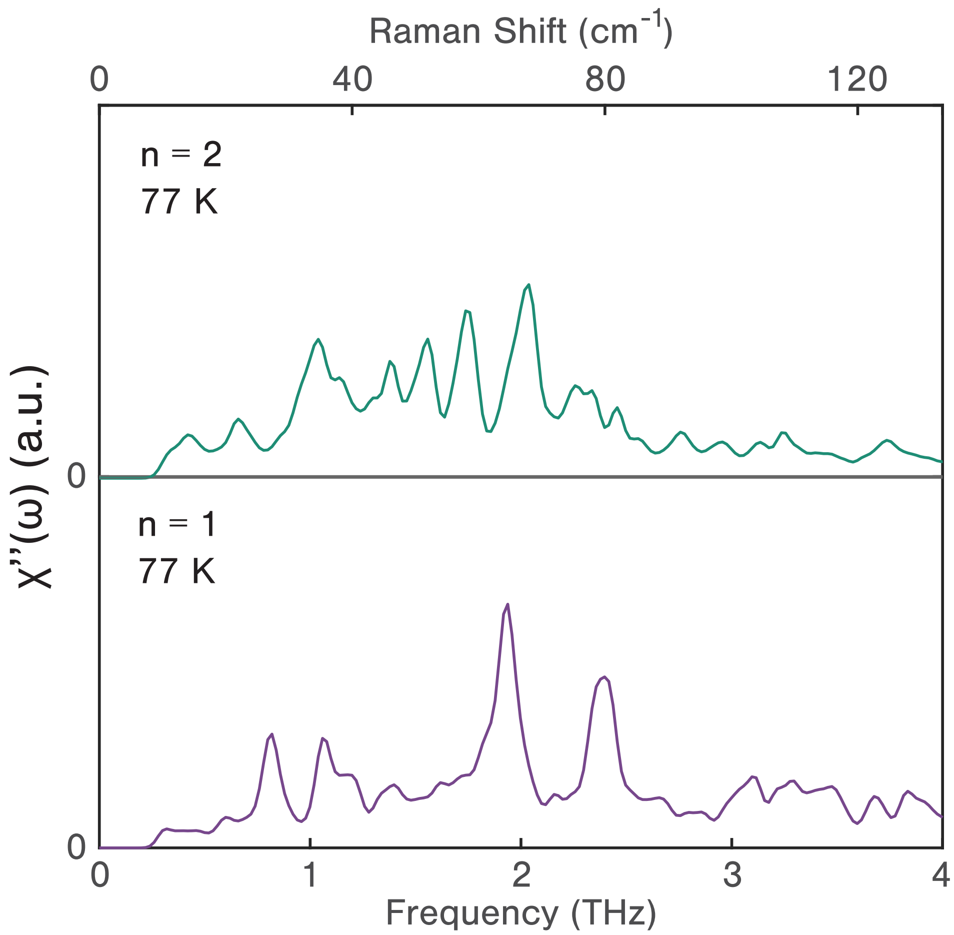

Fig. S9: Simulated Raman spectra with a modified thermal damping time of 0.1 ps. The upper and bottom spectra correspond to n=2 and n=1 2DHPs, respectively.

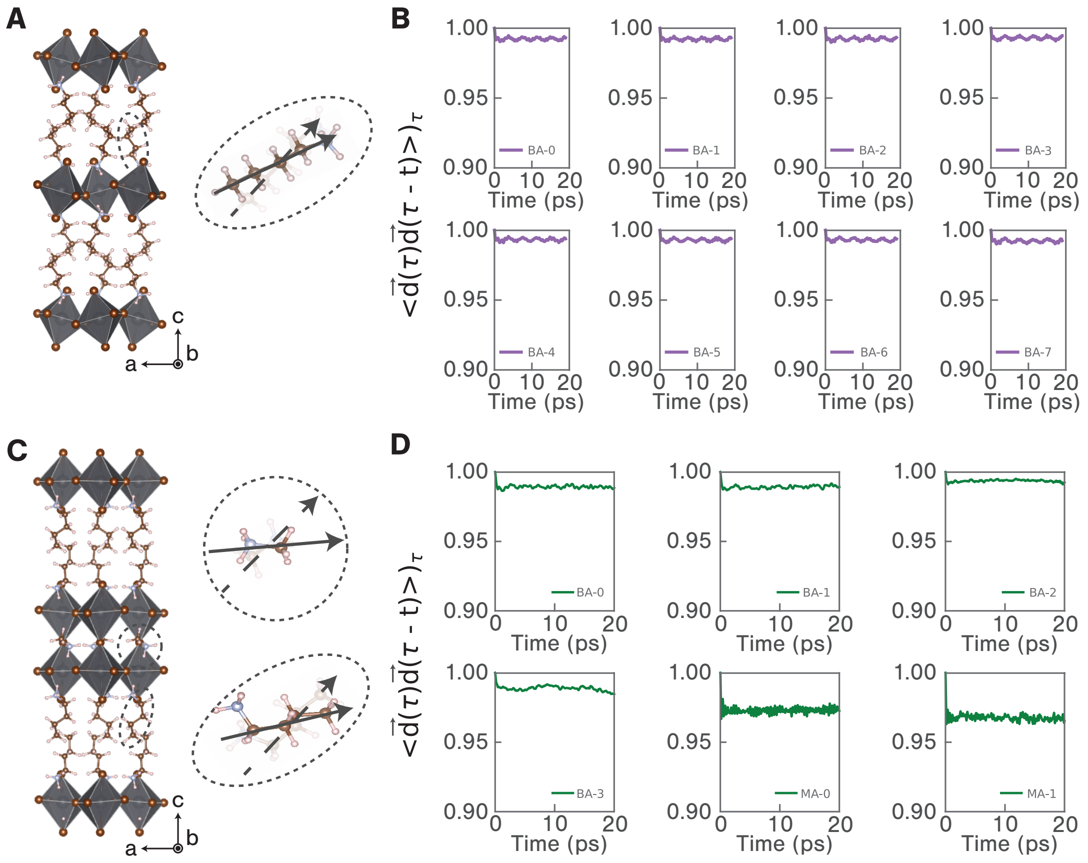

Fig. S10: Time auto-correlation function of organic cation orientations (**A**) Crystal structure of $(BA)_2PbBr_4$, highlighting the reorientation dynamics of the BA cations (indicated by dashed elliptic circle). (**B**) Time auto-correlation functions of six BA cations in the n=1 system. (**C**) Crystal structure of $(BA)_2MAPb_2Br_7$, showing the reorientation dynamics of BA and MA cations (indicated by dashed elliptic and round circles, respectively). (**D**) Time auto-correlation functions of four BA cations and two MA cations in the n=2 2DHP, indicating n=2 2DHP has more disorder compare to n=1 2DHP.

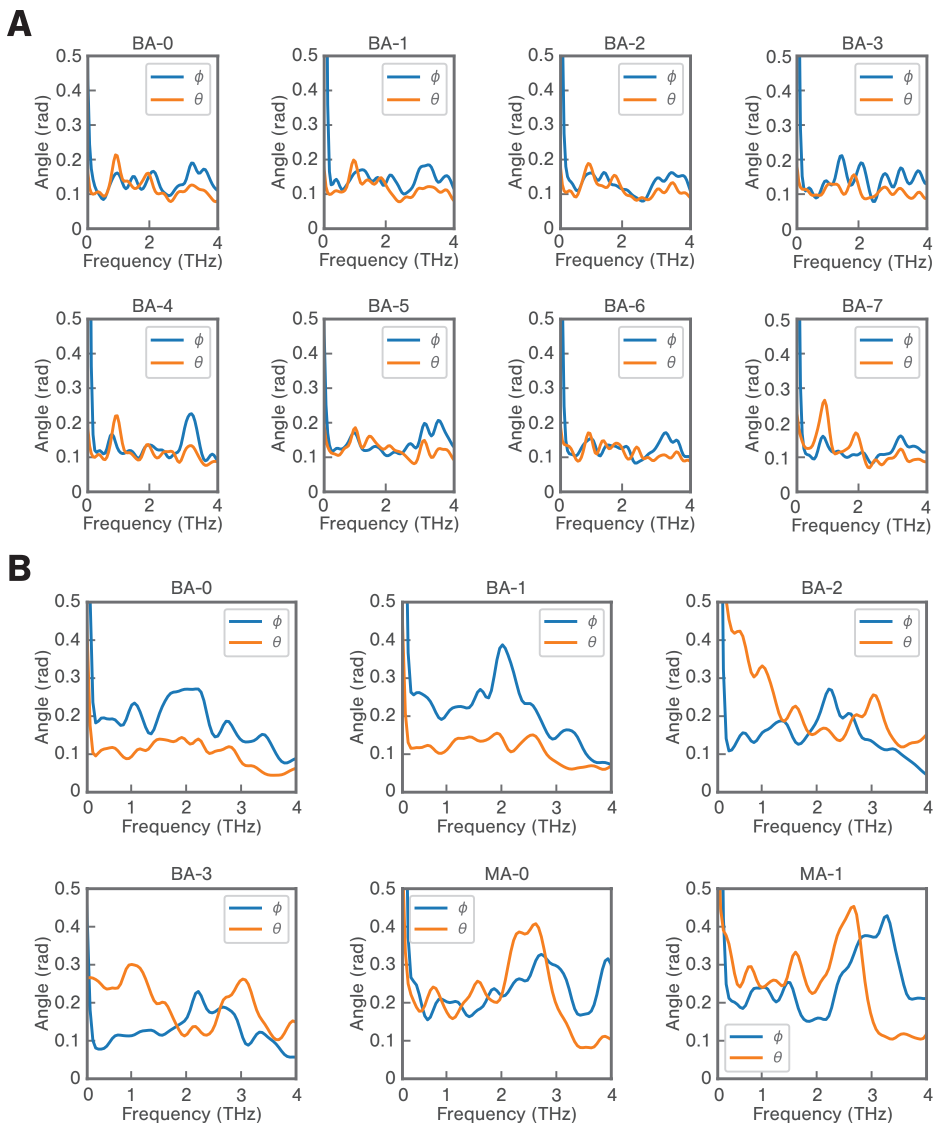

Fig. S11: Origin of structual and dynamical disorder in the n=2 2DHP compared with n=1 2DHP. The reorientation of first C and N bond from MA and BA molecules in spherical coordinates (**A**) For n=1 2DHP, the eight BA molecules has very similar typical frequency indicating they are homogenous everywhere. The neighboring octahedral has spatial equivalent oscillation environment. (**B**) For n=2 2DHP, BA and MA have diffrent oscillation frequency. This indicates Pb-Br octahedral has spatial inequivalent oscillation environment, which contribute to the broading of line shape. The spatial in-equivalence came from local chemical environment difference. The MA molecules are reorienting in the octahedral cage while BA are outside the cage.

Table S1: Reagent quantities used for bromide 2D LHP syntheses

Items	$(BA)_2PbBr_4$	$(BA)_2MAPb_2Br_7$
PbO mass (g)	0.558	0.279
Number of moles of PbO (m*mol*)	2.5	1.25
HBr volume to make $PbBr_2$ solution (mL)	3	1.5
MABr mass (g)	-	0.0700
Number of moles of MABr (m*mol*)	-	0.625
HBr volume to make MABr solution (mL)	-	0.4
BA volume (μL)	247	74
Number of moles of BA (m*mol*)	2.5	0.75
Additional volume of HBr for dilution (mL)	5	0.5
Total volume of HBr used (mL)	8	2.4

Movie S1: Lattice motion corresponding to the 0.7 THz Raman peak in the n=1 2DHP.

Movie S2: Lattice motion corresponding to the 1.1 THz Raman peak in the n=1 2DHP.

Movie S3: Lattice motion corresponding to the 1.5 THz Raman peak in the n=1 2DHP.

Movie S4: Lattice motion corresponding to the 1.8 THz Raman peak in the n=1 2DHP.

Movie S5: Lattice motion corresponding to the 1.8 THz Raman peak in the n=2 2DHP.

REFERENCES AND NOTES

1. X. Gong, O. Voznyy, A. Jain, W. Liu, R. Sabatini, Z. Piontkowski, G. Walters, G. Bappi, S. Nokhrin, O. Bushuyev, M. Yuan, R. Comin, D. M. Camant, S. O. Kelley, E. H. Sargent, Electron–phonon interaction in efficient perovskite blue emitters. *Nat. Mater.* **17**, 550–556 (2018).

2. G. Grancini, M. K. Nazeeruddin, Dimensional tailoring of hybrid perovskites for photovoltaics. *Nat Rev Mater* **4**, 4–22 (2019).

3. M. D. Smith, B. A. Connor, H. I. Karunadasa, Tuning the luminescence of layered halide perovskites. *Chem. Rev.* **119**, 3104–3139 (2019).

4. C. M. Mauck, W. A. Tisdale, Excitons in 2D organic–inorganic halide perovskites. *Trends in Chem.* **1**, 380–393 (2019).

5. X. Li, J. M. Hoffman, M. G. Kanatzidis, The 2D halide perovskite rulebook: How the spacer influences everything from the structure to optoelectronic device efficiency. *Chem. Rev.* **121**, 2230–2291 (2021).

6. E. Shi, Y. Gao, B. P. Finkenauer, A. H. Coffey, L. Dou, Two-dimensional halide perovskite nanomaterials and heterostructures. *Chem. Soc. Rev.* **47**, 6046–6072 (2018).

7. B. Saparov, D. B. Mitzi, Organic-inorganic perovskites: Structural versatility for functional materials design. *Chem. Rev.* **116**, 4558–4596 (2016).

8. W. Paritmongkol, N. S. Dahod, A. Stollmann, N. Mao, C. Settens, S. L. Zheng, W. A. Tisdale, Synthetic variation and structural trends in layered two-dimensional alkylammonium lead halide perovskites. *Chem. Mater.* **31**, 5592–5607 (2019).

9. T. Hang, W. Zhang, H.-Y. Ye, R.-G. Xiong, Metal-organic complex ferroelectrics. *Chem. Soc. Rev.* **40**, 3577–3598 (2011).

10. X. G. Chen, X. J. Song, Z. X. Zhang, P. F. Li, J. Z. Ge, Y. Y. Tang, J. X. Gao, W. Y. Zhang, D. W. Fu, Y. M. You, R. G. Xiong, Two-dimensional layered perovskite ferroelectric with giant piezoelectric voltage coefficient. *J. Am. Chem. Soc.* **142**, 1077–1082 (2019).

11. Y. H. Kim, Y. Zhai, H. Lu, X. Pan, C. Xiao, E. A. Gaulding, S. P. Harvey, J. J. Berry, Z. V. Vardeny, J. M. Luther, M. C. Beard, Chiral-induced spin selectivity enables a room-temperature spin light-emitting diode. *Science* **371**, 1129–1133 (2021).

12. W. Li, Z. Wang, F. Deschler, S. Gao, R. H. Friend, A. K. Cheetham, Chemically diverse and multifunctional hybrid organic–inorganic perovskites. *Nat. Rev. Mater.* **2**, 16099 (2017).

13. M. Menahem, Z. Dai, S. Aharon, R. Sharma, M. Asher, Y. Diskin-Posner, R. Korobko, A. M. Rappe, O. Yaffe, Strongly anharmonic octahedral tilting in two-dimensional hybrid halide perovskites. *ACS Nano* **15**, 10153–10162 (2021).

14. M. Z. Mayers, L. Z. Tan, D. A. Egger, A. M. Rappe, D. R. Reichman, How lattice and charge fluctuations control carrier dynamics in halide perovskites. *Nano Lett.* **18**, 8041–8046 (2018).

15. D. A. Egger, A. Bera, D. Cahen, G. Hodes, T. Kirchartz, L. Kronik, R. Lovrincic, A. M. Rappe, D. R. Reichman, O. Yaffe, What remains unexplained about the properties of halide perovskites? *Adv. Mater.* **30**, 1800691 (2018).

16. K. Miyata, X.-Y. Zhu, Ferroelectric large polarons. *Nat. Mater.* **17**, 379–381 (2018).

17. J. P. Rivett, L. Z. Tan, M. B. Price, S. A. Bourelle, N. J. Davis, J. Xiao, Y. Zou, R. Middleton, B. Sun, A. M. Rappe, D. Credgington, F. Deschler, Long-lived polarization memory in the electronic states of lead-halide perovskites from local structural dynamics. *Nat. Commun.* **9**, 3531 (2018).

18. D. Spirito, Y. Asensio, L. E. Hueso, B. Martín-García, Raman spectroscopy in layered hybrid organic-inorganic metal halide perovskites. *J. Phys. Mater.* **5**, 034004 (2022).

19. B. Dhanabalan, Y. C. Leng, G. Biffi, M. L. Lin, P. H. Tan, I. Infante, L. Manna, M. P. Arciniegas, R. Krahne, Directional anisotropy of the vibrational modes in 2D-layered perovskites. *ACS Nano* **14**, 4689–4697 (2020).

20. J. Hlinka, T. Ostapchuk, D. Nuzhnyy, J. Petzelt, P. Kuzel, C. Kadlec, P. Vanek, I. Ponomareva, L. Bellaiche, Coexistence of the phonon and relaxation soft modes in the terahertz dielectric response of tetragonal BaTiO3. *Phys. Rev. Lett.* **101**, 167402 (2008).

21. O. Yaffe, Y. Guo, L. Z. Tan, D. A. Egger, T. Hull, C. C. Stoumpos, F. Zheng, T. F. Heinz, L. Kronik, M. G. Kanatzidis, J. S. Owen, A. M. Rappe, M. A. Pimenta, L. E. Brus, Local polar fluctuations in lead halide perovskite crystals. *Phys. Rev. Lett.* **118**, 136001 (2017).

22. N. S. Dahod, A. France-Lanord, W. Paritmongkol, J. C. Grossman, W. A. Tisdale, Low-frequency Raman spectrum of 2D layered perovskites: Local atomistic motion or superlattice modes? *J. Chem. Phys.* **153**, 044710 (2020).

23. O. Yaffe, A. Chernikov, Z. M. Norman, Y. Zhong, A. Velauthapillai, A. Van Der Zande, J. S. Owen, T. F. Heinz, Excitons in ultrathin organic-inorganic perovskite crystals. *Phys. Rev. B* **92**, 045414 (2015).

24. J. D. Ziegler, K. Q. Lin, B. Meisinger, X. Zhu, M. Kober-Czerny, P. K. Nayak, C. Vona, T. Taniguchi, K. Watanabe, C. Draxl, H. J. Snaith, J. M. Lupton, D. A. Egger, A. Chernikov, Excitons at the phase transition of 2D hybrid perovskites. *ACS Photonics* **9**, 3609–3616 (2022).

25. C. M. Mauck, A. France-Lanord, A. C. Hernandez Oendra, N. S. Dahod, J. C. Grossman, W. A. Tisdale, Inorganic cage motion dominates excited-state dynamics in 2D-layered perovskites $(C_xH_{2x+1}NH_3)_2PbI_4$ (x = 4–9). *J. Phys. Chem. C* **123**, 27904–27916 (2019).

26. Y.-X. Yan, K. A. Nelson, Impulsive stimulated light scattering. II. Comparison to frequency-domain light-scattering spectroscopy. *J. Chem. Phys.* **87**, 6257–6265 (1987).

27. T. P. Dougherty, G. P. Wiederrecht, K. A. Nelson, M. H. Garrett, H. P. Jensen, C. Warde, Femtosecond resolution of soft mode dynamics in structural phase transitions. *Science* **258**, 770–774 (1992).

28. Q. Zhong, J. T. Fourkas, Optical Kerr effect spectroscopy of simple liquids. *J. Phys. Chem. B.* **112**, 15529–15539 (2008).

29. K. Miyata, D. Meggiolaro, M. T. Trinh, P. P. Joshi, E. Mosconi, S. C. Jones, F. De Angelis, X. Y. Zhu, Large polarons in lead halide perovskites. *Sci. Adv.* **3**, e1701217 (2017).

30. H. Zhu, K. Miyata, Y. Fu, J. Wang, P. P. Joshi, D. Niesner, K. W. Williams, S. Jin, X. Y. Zhu, Screening in crystalline liquids protects energetic carriers in hybrid perovskites. *Science* **353**, 1409–1413 (2016).

31. S. F. Maehrlein, P. P. Joshi, L. Huber, F. Wang, M. Cherasse, Y. Liu, D. M. Juraschek, E. Mosconi, D. Meggiolaro, F. De Angelis, X.-Y. Zhu, Decoding ultrafast polarization responses in lead halide perovskites by the two-dimensional optical Kerr effect. *Proc. Natl. Acad. Sci.* **118**, e2022268118 (2021).

32. F. Thouin, D. A. Valverde-Chávez, C. Quarti, D. Cortecchia, I. Bargigia, D. Beljonne, A. Petrozza, C. Silva, A. R. Srimath Kandada, Phonon coherences reveal the polaronic character of excitons in two-dimensional lead halide perovskites. *Nat. Mater.* **18**, 349–356 (2019).

33. P. Guo, Y. Xia, J. Gong, D. H. Cao, X. Li, X. Li, Q. Zhang, C. C. Stoumpos, M. S. Kirschner, H. Wen, V. B. Prakapenka, J. B. Ketterson, A. B. F. Martinson, T. Xu, M. G. Kanatzidis, M. K. Y. Chan, R. D. Schaller, Direct observation of bandgap oscillations induced by optical phonons in hybrid lead iodide perovskites. *Adv. Funct. Mater.* **30**, 1907982 (2020).

34. L. N. Quan, Y. Park, P. Guo, M. Gao, J. Jin, J. Huang, J. K. Copper, A. Schwartzberg, R. Schaller, D. T. Limmer, P. Yang, Vibrational relaxation dynamics in layered perovskite quantum wells. *Proc. Natl. Acad. Sci. U.S.A.* **118**, e2104425118 (2021).

35. J. Fu, M. Li, A. Solanki, Q. Xu, Y. Lekina, S. Ramesh, Z. X. Shen, T. C. Sum, Electronic states modulation by coherent optical phonons in 2D halide perovskites. *Adv. Mater.* **33**, 2006233 (2021).

36. M. C. Hoffmann, N. C. Brandt, H. Y. Hwang, K.-L. Yeh, K. A. Nelson, Terahertz Kerr effect. *Appl. Phys. Lett.* **95**, 231105 (2009).

37. A. A. Melnikov, V. E. Anikeeva, O. I. Semenova, S. V. Chekalin, Terahertz Kerr effect in a methylammonium lead bromide perovskite crystal. *Phys. Rev. B* **105**, 174304 (2022).

38. L. Huber, S. F. Maehrlein, F. Wang, Y. Liu, X. Y. Zhu, The ultrafast Kerr effect in anisotropic and dispersive media. *J. Chem. Phys.* **154**, 094202 (2021).

39. M. Frenzel, M. Cherasse, J. M. Urban, F. Wang, B. Xiang, L. Nest, L. Huber, L. Perfetti, M. Wolf, T. Kampfrath, X. Y. Zhu, S. F. Maehrlein, Nonlinear THz control of the lead halide perovskite lattice. arXiv:2301.03508 [cond-mat.mtrl-sci] (2023).

40. H. Elgabarty, T. Kampfrath, D. J. Bonthuis, V. Balos, N. K. Kaliannan, P. Loche, R. R. Netz, M. Wolf,

T. D. Kuhne, M. Sajadi, Energy transfer within the hydrogen bonding network of water following resonant terahertz excitation. *Sci. Adv.* **6**, eaay7074 (2020).

41. H. Zhao, Y. Tan, L. Zhang, R. Zhang, M. Shalaby, C. Zhang, Y. Zhao, X. Zhang, Ultrafast hydrogen bond dynamics of liquid water revealed by terahertz-induced transient birefringence. *Light Sci. Appl.* **9**, 136 (2020).

42. G. Khalsa, N. A. Benedek, J. Moses, Ultrafast control of material optical properties via the infrared resonant Raman effect. *Phys. Rev. X* **11**, 021067 (2021).

43. M. Först, C. Manzoni, S. Kaiser, Y. Tomioka, Y. Tokura, R. Merlin, A. Cavalleri, Nonlinear phononics as an ultrafast route to lattice control. *Nat. Phys.* **7**, 854–856 (2011).

44. D. M. Juraschek, S. F. Maehrlein, Sum-frequency ionic Raman scattering. *Phys. Rev. B* **97**, 174302 (2018).

45. C. Aku-Leh, J. Zhao, R. Merlin, J. Menendez, M. Cardona, Long-lived optical phonons in ZnO studied with impulsive stimulated Raman scattering. *Phys. Rev. B* **71**, 205211 (2005).

46. C. L.Johnson, B. E. Knighton, J. A. Johnson, Distinguishing nonlinear terahertz excitation pathways with two-dimensional spectroscopy. *Phys. Rev. Lett.* **122**, 073901 (2019).

47. S. Maehrlein, A. Paarmann, M. Wolf, T. Kampfrath, Terahertz sum-frequency excitation of a Raman-active phonon. *Phys. Rev. Lett.* **119**, 127402 (2017).

48. Y.-X. Yan, E. B. Gamble Jr, K. A. Nelson, Impulsive stimulated scattering: General importance in femtosecond laser pulse interactions with matter, and spectroscopic applications. *J. Chem. Phys.* **83**, 5391–5399 (1985).

49. A. Maradudin, R. Wallis, Ionic Raman effect. I. Scattering by localized vibration modes. *Phys. Rev. B* **2**, 4294–4299 (1970).

50. R. Wallis, A. Maradudin, Ionic Raman effect. II. The first-order ionic Raman effect. *Phys. Rev. B* **3**, 2063 (1971).

51. L. Humphreys, Ionic raman effect. III. First- and second-order ionic Raman effects. *Phys. Rev. B* **6**, 3886–3897 (1972).

52. X. Li, T. Qiu, J. Zhang, E. Baldini, J. Lu, A. M. Rappe, K. A. Nelson, Terahertz field–induced ferroelectricity in quantum paraelectric $SrTiO_3$. *Science* **364**, 1079–1082 (2019).

53. A. von Hoegen, M. Fechner, M. Forst, N. Taherian, E. Rowe, A. Ribak, J. Porras, B. Keimer, M. Michael, E. Demler, A. Cavalleri, Amplification of superconducting fluctuations in driven $YBa_2Cu_3O_{6+x}$. *Phys. Rev. X* **12**, 031008 (2022).

54. G. Mead, H.-W. Lin, I.-B. Magdau, T. F. Miller III, Geoffrey A. Blake, Sum-frequency signals in 2D-terahertz-terahertz-Raman spectroscopy. *J. Phys. Chem. B.* **124**, 8904—8908 (2020).

55. M. A. Reyes-Martinez, P. Tan, A. Kakekhani, S. Banerjee, A. A. Zhumekenov, W. Peng, O. M. Bakr, A. M. Rappe, Y.-L. Loo, Unraveling the elastic properties of (quasi) two-dimensional hybrid perovskites: A joint experimental and theoretical study. *ACS Appl. Mater. Interfaces* **12**, 17881–17892 (2020).

56. X. Gonze, Adiabatic density-functional perturbation theory. *Phys. Rev. A* **52**, 1096–1114 (1995).

57. S. Baroni, P. Giannozzi, A. Testa, Green's-function approach to linear response in solids. *Phys. Rev. Lett.* **58**, 1861 (1987), 1864.

58. S. Baroni, S. De Gironcoli, A. Dal Corso, P. Giannozzi, Phonons and related crystal properties from density-functional perturbation theory. *Rev. Mod. Phys.* **73**, 515 (2001), 562.

59. A. S. Disa, T. F. Nova, A. Cavalleri, Engineering crystal structures with light. *Nat. Phys.* **17**, 1087–1092 (2021).

60. G. Grinblat, I. Abdelwahab, M. P. Nielsen, P. Dichtl, K. Leng, R. F. Oulton, K. P. Loh, S. A. Maier, Ultrafast all-optical modulation in 2D hybrid perovskites. *ACS Nano* **13**, 9504–9510 (2019).

61. Z. Sun, A. Martinez, F. Wang, Optical modulators with 2D layered materials. *Nat. Photonics* **10**, 227–238 (2016).

62. C. C. Stoumpos, D. H. Cao, D. J. Clark, J. Young, J. M. Rondinelli, J. I. Jang, J. T. Hupp, M. G. Kanatzidis, Ruddlesden–Popper hybrid lead iodide perovskite 2D homologous semiconductors. *Chem. Mater.* **28**, 2852–2867 (2016).

63. K.-L. Yeh, J. Hebling, M. C. Hoffmann, K. A. Nelson, Generation of high average power 1 kHz shaped THz pulses via optical rectification. *Opt. Commun.* **281**, 3567–3570 (2008).

64. F. Y. Gao, Z. Zhang, Z.-J. Liu, K. A. Nelson, High-speed two-dimensional terahertz spectroscopy with echelon-based shot-to-shot balanced detection. *Opt. Lett.* **47**, 3479–3482 (2022).

65. J. P. Perdew, K. Burke, M. Ernzerhof, Generalized gradient approximation made simple. *Phys. Rev. Lett.* **77**, 3865–3868 (1996).

66. A. M. Rappe, K. M. Rabe, E. Kaxiras, J. D. Joannopoulos, Optimized pseudopotentials. *Phys. Rev. B* **41**, 1227–1230 (1990).

67. Opium-pseudopotential generation project; http://opium.sourceforge.net.

68. M. Thomas, M. Brehm, R. Fligg, P. Vöhringer, B. Kirchner, Computing vibrational spectra from ab initio molecular dynamics. *Phys. Chem. Chem. Phys.* **15**, 6608–6622 (2013).

69. R. Sharma, Z. Dai, L. Gao, T. M. Brenner, L. Yadgarov, J. Zhang, Y. Rakita, R. Korobko, A. M. Rappe, O. Yaffe, Elucidating the atomistic origin of anharmonicity in tetragonal $CH_3NH_3PbI_3$ with Raman scattering. *Phys. Rev. Mater.* **4**, 092401 (2020).

70. R. Sharma, M. Menahem, Z. Dai, L. Gao, T. M. Brenner, L. Yadgarov, J. Zhang, Y. Rakita, R. Korobko, I. Pinkas, A. M. Rappe, O. Yaffe, Lattice mode symmetry analysis of the orthorhombic phase of methylammonium lead iodide using polarized Raman. *Phys. Rev. Mater.* **4**, 051601 (2020).

71. N. Wiener, Generalized harmonic analysis. *Acta Math.* **55**, 117–258 (1930).

72. A. Khintchine, Korrelationstheorie der stationären stochastischen prozesse. *Math. Ann.* **109**, 604–615 (1934).

73. P. Giannozzi, S. Baroni, N. Bonini, M. Calandra, R. Car, C. Cavazzoni, D. Ceresoli, G. L. Chiarotti, M.

Cococcioni, QUANTUM ESPRESSO: A modular and open-source software project for quantum simulations of materials. *J. Phys. Condens. Matter* **21**, 395502 (2009).

74.P. Giannozzi, O. Andreussi, T. Brumme, O. Bunau, M. B. Nardelli, M. Calandra, R. Car, C. Cavazzoni, D. Ceresoli, M. Cococcioni, Advanced capabilities for materials modelling with Quantum ESPRESSO. *J. Phys. Condens. Matter* **29**, 465901 (2017).

75. S. Nosé, A unified formulation of the constant temperature molecular dynamics methods. *J. Chem. Phys.* **81**, 511–519 (1984).

76. W. G. Hoover, Canonical dynamics: Equilibrium phase-space distributions. *Phys. Rev. A* **31**, 1695 (1985), 1697.

77. L. Li, X. Liu, Y. Li, Z. Xu, Z. Wu, S. Han, K. Tao, M. Hong, J. Luo, Z. Sun, Two-dimensional hybrid perovskite-type ferroelectric for highly polarization-sensitive shortwave photodetection. *J. Am. Chem. Soc.* **141**, 2623–2629 (2019).

78. L. Dou, A. B. Wong, Y. Yu, M. Lai, N. Kornienko, S. W. Eaton, A. Fu, C. G. Bischak, J. Ma, T. Ding, N. S. Ginsberg, L. Wang, A. P. Alivisatos, P. Yang, Atomically thin two-dimensional organic-inorganic hybrid perovskites. *Science* **349**, 1518—1521 (2015).

79. M. Hase, K. Mizoguchi, H. Harima, S.-i. Nakashima, K. Sakai, Dynamics of coherent phonons in bismuth generated by ultrashort laser pulses. *Phys. Rev. B* **58**, 5448–5452 (1998).

80. A. P. Thompson, H. M. Aktulga, R. Berger, D. S. Bolintineanu, W. M. Brown, P. S. Crozier, P. J. in 't Veld, A. Kohlmeyer, S. G. Moore, T. D. Nguyen, R. Shan, M. J. Stevens, J. Tranchida, C. Trott, S. J. Plimpton, Lammps - a flexible simulation tool for particle-based materials modeling at the atomic, meso, and continuum scales. *Comput. Phys. Commun.* **271**, 108171 (2022).

81. V. Kumar, M. Casella, E. Molotokaite, D. Gatti, P. Kukura, C. Manzoni, D. Polli, M. Marangoni, G. Cerullo, Balanced-detection Raman-induced Kerr-effect spectroscopy. *Phys. Rev. A* **86**, 053810 (2012).
